# Characteristics and primary care experiences of people who self-report as autistic: a probability sample survey of adults registered with primary care services in England

**DOI:** 10.1136/bmjopen-2023-081388

**Published:** 2024-09-13

**Authors:** Samuel Joseph Tromans, Lucy Teece, Catherine Saunders, Sally McManus, Traolach Brugha

**Affiliations:** 1 Department of Population Health Sciences, University of Leicester, Leicester, UK; 2 Adult Learning Disability Service, Leicestershire Partnership NHS Trust, Leicester, UK; 3 Department of Psychiatry, University of Cambridge, Cambridge, UK; 4 School of Health and Psychological Sciences, City University of London, London, UK; 5 National Centre for Social Research, London, UK; 6 Adult Autism Assessment Service, Leicestershire Partnership NHS Trust, Leicester, UK

**Keywords:** Primary Health Care, Health Services, PUBLIC HEALTH, Adult psychiatry, EPIDEMIOLOGY

## Abstract

**Abstract:**

**Objectives:**

Little is known about adults who self-report as autistic. This study aimed to profile the demographic characteristics, long-term health conditions and primary care experiences of adults who self-report as autistic (including those with and without a formal diagnosis).

**Design/setting:**

A nationally representative cross-sectional survey of adults registered with National Health Service (NHS) General Practitioner (GP) surgeries in England.

**Participants:**

623 157 survey respondents aged 16 and over, including 4481 who self-report as autistic.

**Outcomes:**

Weighted descriptive statistics, with 95% CIs. Logistic regression modelling adjusted for age, gender, ethnicity and area-level deprivation compared those who self-report as autistic with the rest of the population.

**Results:**

A total of 4481 of the 623 157 survey participants included in the analysis self-reported autism, yielding a weighted proportion estimate of 1.41% (95% CI 1.35% to 1.46%). Adults self-reporting as autistic were more likely to be younger, male or non-binary, to identify as a gender different from their sex at birth, have a non-heterosexual sexual identity, be of white or mixed or multiple ethnic groups, non-religious, without caring responsibilities, unemployed, live in more deprived areas and not smoke. All chronic conditions covered were more prevalent among adults self-reporting as autistic, including learning disability, mental health conditions, neurological conditions, dementia, blindness or partial sight and deafness or hearing loss. Adults self-reporting as autistic were also less likely to report a positive experience of making an appointment (adjusted OR (aOR) 0.90, 95% CI 0.82 to 0.98) and navigating GP practice websites (aOR 0.78, 95% CI 0.70 to 0.87) and more likely to report seeking advice from a friend or family member prior to making an appointment (aOR 1.25, 95% CI 1.14 to 1.38) and having a preferred GP (aOR 2.25, 95% CI 2.06 to 2.46). They were less likely to report that their needs were met (aOR 0.73, 95% CI 0.65 to 0.83).

**Conclusions:**

Adults self-reporting as autistic have a distinctive sociodemographic profile and heightened rates of long-term conditions. They report challenges in both accessing primary care and having their needs met when they do. These findings should inform future care initiatives designed to meet the needs of this group.

**STRENGTHS AND LIMITATIONS OF THIS STUDY**

- This study had a large sample size, with 4481 adults self-reporting as autistic, and a comparator group of 623 157 adults who did not self-report as autistic.
- The data described are self-reported by patients, providing insights into their subjective experiences of primary care.
- The data are cross-sectional in nature, and longitudinal data are needed to better understand causality.
- Patients self-reported their long-term conditions, with no means to compare their responses against their formal diagnoses on their medical records.

## Introduction

 Autism is defined as a lifelong neurodevelopmental condition, characterised by impairments in social communication, interaction and a repertoire of restricted repetitive behaviours.^1^ Autism has an estimated prevalence of around 1% among community-based adults in England,^2^ with increased numbers among adults using mental health services.^3 4^ Clinically diagnosed autistic people have been found to be at increased risk of a range of chronic health conditions compared with their non-autistic peers,^5^ as well as premature mortality.^6^ Furthermore, diagnosed autistic people in the UK have significantly greater mortality rates compared with people without an autism diagnosis; 1.71 (95% CI 1.39 to 2.11) times the general population for people diagnosed with autism and not intellectual disability, and 2.83 (95% CI 2.33 to 3.43) times for people diagnosed with both autism and intellectual disability.^7^ Relatedly, the life expectancy for people diagnosed with autism but not intellectual disability was found to be shorter by 6.14 years (95% CI 2.84 to 9.07) for men and 6.45 years (95% CI 1.37 to 11.57) for women.^7^ All autistic people will access primary care during their lives, and for most health problems, the journey to diagnosis and treatment begins in this setting.^8^ Thus, it is essential that primary care services are comprehensively meeting the needs of the autistic community.

Accordingly, autism was recognised as a priority within the National Health Service (NHS) Long Term Plan,^9^ with goals set to address the causes of morbidity and preventable deaths in this group, as well as improve understanding across the NHS with regard to the needs of autistic people. Furthermore, in 2021, the UK government published a national strategy for autistic people,^10^ with the aim to improve the lives of autistic people, as well as their families and carers. Adult general population surveys suggest that most adults with autism do not have a clinical diagnosis officially recorded in their medical records^2^ Therefore, both those diagnosed autistic people and those who regard themselves to be autistic but do not currently have a diagnosis merit studying. However, there is a lack of autism research focused on adults, with a Web of Science topic search demonstrating that in 2020, only around 21% of autism publications contained that ‘adult*’ search term.^11^ Research with adults diagnosed with autism indicates that they may face a range of social and economic inequalities, such as low employment rates.^12^ However, it is less clear to what extent these inequalities extend to the broader group of people who self-report as being autistic.

Among adults who regard themselves as autistic, some have a clinical diagnosis confirmed via assessment by a healthcare professional and some have not. There are numerous barriers to obtaining a formal autism diagnostic assessment, including a lack of autism awareness among healthcare professionals, lengthy waiting lists and private assessments, costs.^13^ Furthermore, a perceived lack of postdiagnostic support may discourage patients and their primary care physician from seeking such an assessment.^13^ Levels of autism underdiagnosis appear to be greater in older age groups, with O’Nions *et al*^14^ estimating that over 90% of autistic adults of 50 years and older are undiagnosed. In addition, many people may consider themselves to be autistic but would not meet diagnostic thresholds if assessed for autism, although they may have other significant, unidentified mental health conditions. This may be in part due to autistic features overlapping with those of other neurodevelopmental conditions, such as learning disability and attention deficit hyperactivity disorder.^15^ Additionally, it is important to recognise the limitations of current autism diagnostic tools, particularly in certain groups, such as women and girls.^16^

The General Practice Patient Survey (GPPS), conducted by Ipsos MORI, is an NHS England-funded annual national cross-sectional survey of adults aged 16 and over who have been registered with a General Practitioner (GP) practice in England for at least 6 months, to provide evidence to support healthcare improvement for process measures of care quality. The questionnaire covers patient experiences of primary care, as well as participants’ demographic characteristics and health and socioeconomic circumstances.^17^ The scale, health and socioeconomic characteristics, and primary care experiences of adults in England who report that they are autistic have rarely been considered. Understanding the needs of the wider population reporting that they are autistic, rather than solely those with a clinical diagnosis or those identified in population surveys, is essential to anticipating demand for assessment services, and for understanding how current models of healthcare do and do not meet their needs particularly for assessment. In this analysis of the 2022 GPPS, we report findings relating to adults who self-report as autistic, with this group defined as survey respondents who, in response to the survey question item ‘which, if any, of the following long-term conditions do you have?’, selected the checklist option ‘autism or autism spectrum condition.’^18^ Our research question was ‘what are the demographic characteristics, long-term health conditions, and primary care experiences of adults who self-report as autistic in England?’

## Methods

### GPPS 2022 survey sampling

This study uses data collected for the 2022 GPPS, covering patients registered with GP practices in England from 10 January 2022 to 11 April 2022.^19^ Patients aged 16 years and over (hereafter referred to as ‘adults’) with a valid NHS number who had been continuously registered with an NHS GP practice in England for at least 6 months were randomly selected for contact.^19^ With respect to patient consent, the voluntary nature of the GPPS was explained online, with an option for patients to opt-out.^20^ Over 2.47 million questionnaires were sent out, and 719 137 completed questionnaires were returned, representing a 29.1% national response rate.^17^ The sample was designed to achieve at least 100 responses per GP practice and 200 responses per Primary Care Network.^19^ A weighting scheme was developed by the survey provider (Ipsos MORI) to correct for sampling design and reduce the impact of non-responders and improve the representativeness of the analyses to better reflect the GP-registered population.^17 19^ NHS primary care records were accessed only to identify the sample of people to be invited to take part and to extract their postal address; no other NHS records, such as recorded diagnoses were accessed. Further methodological details for GPPS 2022, as well as the 2022 questionnaire, can be found in a published technical annex.^19^ Patient-level data for GPPS 2022 were shared with the authors according to a data-sharing agreement with NHS England.

### Self-reported autism identification

The option ‘autism or autism spectrum condition’ as a response to the multiple-choice question ‘which, if any, of the following long-term conditions do you have?’ was introduced in the 2019 survey^21^ and has remained for its subsequent iterations. This item was used to differentiate between adults with self-reported autism and adults who did not self-report autism and provides the novel opportunity to study in greater detail this self-identifying population. These two groups are summarised in table 1. There were no means to identify which participants had a formal diagnosis of autism on their medical records (both among those self-reporting and not self-reporting autism); this issue is discussed in further detail in the Strengths and weaknesses of the study subsection of the Discussion. Participants who did not provide a valid response to this question were excluded from the analyses.

**Table 1:** Summary of members of self-reporting as autistic and non-self-reporting as autistic participant groups

Self-reporting as autistic	Not self-reporting as autistic
Clinically diagnosed autistic people who identify as autistic Adults without a clinical diagnosis of autism who identify as autistic and who would meet autism diagnostic criteria if subjected to clinical assessment Adults without a clinical diagnosis of autism who identify as autistic and who would not meet autism diagnostic criteria if subjected to clinical assessment	Clinically diagnosed autistic people who do not identify as autistic Adults without a clinical diagnosis of autism who do not identify as autistic and who would not meet autism diagnostic criteria if subjected to clinical assessment Adults without a clinical diagnosis of autism who do not identify as autistic and who would meet autism diagnostic criteria if subjected to clinical assessment

There were many other long-term conditions included as multiple-choice options with respect to the long-term condition survey question, listed in full in the subsequent subsection of the Methods. Another long-term condition that patients were asked if they identified as having was a learning disability. Considering this, it is possible that some surveys, particularly from those with a learning disability, may have been completed with support from a carer; however, no data were collected with respect to whether any surveys were completed by either assistance from a proxy, or the proxy completing the survey on behalf of the patient.

### Demographic and health measures

Demographic variables included gender, transgender history, sexual identity, age, ethnicity, religion, caring responsibilities and smoking status (see table 2 for how these variables were categorised). Socioeconomic circumstances were captured with two items: employment status and neighbourhood deprivation according to the Index of Multiple Deprivation (IMD) quintile based on the participant’s postcode.

**Table 2:** Demographic characteristics of responders to the 2022 GPPS England, by whether self-report as autistic

Characteristics	Self-reported autistic (yes)N=4481			Self-reported autistic (no)N=618 676			Comparison, percentage point difference	
	N	Unweighted % weighted %*	95% CI	N	Unweighted % weighted %*	95% CI	Weightedppd	95% CI
Gender	4481	100.0		618 676	100.0			
Female	1837	30.2	(28.6, 32)	354 973	52.1	(51.9, 52.3)	−21.8	(−23.5, −20.1)
Male	2455	64.9	(63, 66.7)	261 530	47.4	(47.2, 47.6)	+17.4	(15.6, 19.3)
Non-binary	145	3.9	(3.1, 4.9)	1141	0.3	(0.3, 0.3)	+3.6	(2.8, 4.5)
Prefer to self-describe	44	1.0	(0.7, 1.5)	1032	0.2	(0.2, 0.2)	+0.8	(0.4, 1.2)
Gender matches sex registered at birth	4347	97.0		611 249	98.8			
Yes	4129	93.5	(92.3, 94.6)	608 372	99.4	(99.4, 99.4)	−5.9	(−7.0, −4.8)
No (transgender)	218	6.5	(5.4, 7.7)	2877	0.6	(0.6, 0.6)	+5.9	(4.8, 7.0)
Sexual identity	3933	87.8		584 018	94.4			
Heterosexual or straight	3104	76.4	(74.5, 78.2)	562 678	94.6	(94.5, 94.7)	−18.2	(-20.0, −16.3)
Gay or lesbian	221	6.8	(5.7, 8.0)	9254	2.4	(2.4, 2.5)	+4.3	(3.2, 5.5)
Bisexual	365	10.2	(9.0, 11.6)	6878	2.0	(1.9, 2.0)	+8.3	(7.0, 9.6)
Other	243	6.6	(5.6, 7.7)	5208	1.1	(1.0, 1.1)	+5.5	(4.5, 6.6)
Age	4481	100.0		618 676	100.0			
16–24	1032	35.5	(33.4, 37.6)	21 415	8.8	(8.7, 8.9)	+26.7	(24.6, 28.8)
25–34	1014	31.4	(29.5, 33.4)	47 976	16.6	(16.4, 16.7)	+14.9	(12.9, 16.8)
35–44	768	15.5	(14.2, 16.9)	72 343	17.7	(17.5, 17.8)	−2.2	(−3.5, −0.8)
45–54	647	9.0	(8.2, 9.9)	98 385	17.6	(17.5, 17.7)	−8.6	(−9.5, −7.7)
55–64	552	5.5	(4.9, 6.1)	134 695	16.6	(16.5, 16.7)	−11.1	(-11.8, −10.5)
65–74	282	1.8	(1.5, 2.1)	135 523	12.3	(12.3, 12.4)	−10.5	(-10.8, −10.3)
75 or over	186	1.3	(1.1, 1.6)	108 339	10.4	(10.3, 10.5)	−9.1	(−9.3, −8.8)
Ethnicity	4481	100.0		618 676	100.0			
White	3769	87.7	(86.4, 88.8)	521 125	82.4	(82.3, 82.6)	+5.2	(4.0, 6.5)
Mixed or multiple ethnic groups	155	3.1	(2.6, 3.9)	8800	2.0	(1.9, 2.0)	+1.2	(0.5, 1.8)
Asian or Asian British	315	5.1	(4.4, 6.0)	54 361	9.7	(9.6, 9.8)	−4.6	(−5.3, −3.8)
Black, Black British, Caribbean or African	157	2.7	(2.1, 3.5)	23 180	3.9	(3.8, 3.9)	−1.1	(−1.8, −0.5)
Other ethnic group	85	1.3	(1.0, 1.8)	11 210	2.1	(2.0, 2.1)	−0.7	(−1.1, −0.3)
Religion	4211	94.0		597 112	96.5			
No religion	1976	53.6	(51.5, 55.7)	178 446	37.8	(37.7, 38.0)	+15.7	(13.6, 17.9)
Buddhist	50	1.1	(0.7, 1.7)	4053	0.7	(0.7, 0.8)	+0.4	(−0.1, 0.8)
Christian	1608	34.5	(32.5, 36.5)	353 325	50.4	(50.2, 50.6)	−15.9	(-17.9, −13.9)
Hindu	52	0.8	(0.5, 1.2)	12 292	2.1	(2.0, 2.2)	−1.3	(−1.6, −1.0)
Jewish	41	0.8	(0.5, 1.2)	3511	0.5	(0.5, 0.5)	+0.3	(0.0, 0.6)
Muslim	235	3.7	(3.1, 4.4)	30 938	5.9	(5.8, 6.0)	−2.2	(−2.9, −1.6)
Sikh	18	0.2	(0.1, 0.3)	5630	0.9	(0.8, 0.9)	−0.7	(−0.8, −0.6)
Other	231	5.4	(4.5, 6.3)	8917	1.7	(1.6, 1.7)	+3.7	(2.8, 4.6)
Parental responsibility for child in household	4425	98.7		613 916	99.2			
Yes	656	12.2	(11.1, 13.4)	111 864	25.0	(24.9, 25.2)	−12.8	(−14, −11.7)
No	3769	87.8	(86.6, 88.9)	502 052	75.0	(74.8, 75.1)	+12.8	(11.7, 14.0)
Caring responsibilities due to health or old age	4378	97.7		605 321	97.8			
No	3301	79.4	(77.8, 80.9)	480 917	81.3	(81.1, 81.4)	−1.9	(−3.4, −0.3)
Yes, 1–9 hours/week	432	9.4	(8.3, 10.6)	62 544	9.6	(9.5, 9.7)	−0.3	(−1.4, 0.9)
Yes, 10–49 hours/week	327	5.9	(5.1, 6.9)	34 842	5.3	(5.3, 5.4)	+0.6	(−0.3, 1.5)
Yes, 50+ hours/week	318	5.3	(4.5, 6.1)	27 018	3.7	(3.7, 3.8)	+1.5	(0.7, 2.3)
Employment status	4284	95.6		601 915	97.3			
Full-time work	1040	24.6	(22.9, 26.5)	207 988	46.4	(46.2, 46.6)	−21.7	(-23.5, −19.9)
Part-time work	436	9.9	(8.7, 11.3)	75 264	12.5	(12.4, 12.6)	−2.6	(−3.8, 1.3)
Full-time education	517	17.0	(15.4, 18.8)	12 158	4.6	(4.5, 4.7)	+12.4	(10.7, 14.1)
Unemployed	526	13.6	(12.2, 15.1)	19 920	4.0	(3.9, 4.1)	+9.6	(8.1, 11.1)
Permanently sick/disabled	1021	24.1	(22.3, 25.9)	28 642	4.4	(4.3, 4.4)	+19.7	(17.9, 21.5)
Retired	362	2.6	(2.3, 3.0)	213 545	20.8	(20.7, 20.9)	−18.2	(−18.6, 17.8)
Looking after family/home	169	3.0	(2.5, 3.7)	27 845	4.5	(4.5, 4.6)	−1.5	(−2.1, −0.9)
Other	213	5.1	(4.3, 6.1)	16 553	2.8	(2.7, 2.8)	+2.4	(1.4, 3.3)
Neighbourhood deprivation	4481	100.0		618 676	100.0			
1—Most deprived	1310	28.9	(27.0, 30.9)	121 075	20.2	(20.0, 20.3)	+8.7	(6.8, 10.6)
2	1006	22.8	(21.2, 24.6)	123 769	20.7	(20.5, 20.8)	+2.2	(0.4, 3.9)
3	834	18.4	(16.8, 20.1)	127 736	20.2	(20.1, 20.4)	−1.8	(−3.4, −0.2)
4	750	16.6	(15.2, 18.1)	126 605	19.7	(19.6, 19.9)	−3.2	(−4.6, −1.7)
5—Least deprived	581	13.3	(12.0, 14.6)	119 491	19.2	(19.1, 19.3)	−5.9	(−7.3, −4.6)
Smoking status	4437	99.0		614 542	99.3			
Never smoked	2868	70.7	(68.9, 72.4)	348 450	59.5	(59.3, 59.7)	+11.2	(9.4, 13.0)
Ex-smoker	873	13.8	(12.6, 15.1)	194 017	26.8	(26.6, 26.9)	−13.0	(-14.2, −11.7)
Occasional smoker	320	7.2	(6.2, 8.4)	32 820	6.7	(6.6, 6.8)	+0.6	(−0.5, 1.6)
Regular smoker	376	8.3	(7.3, 9.4)	39 255	7.1	(7.0, 7.2)	+1.2	(0.1, 2.3)

* Unweighted percentages show proportion of non-missing responses; Weighted percentages are calculated using survey design and non-response weights by age, gender, geographical location, and GP practice.

GPGeneral PractitionerGPPSGeneral Practice Patient Surveyppdpercentage point difference

Long-term health conditions listed as checklist options to the multiple-choice question included: Alzheimer’s disease or other cause of dementia; arthritis or ongoing problem with back or joints; blindness or partial sight; a breathing condition such as asthma or chronic obstructive pulmonary disease; cancer (diagnosis or treatment in the last 5 years); deafness or hearing loss; diabetes; a heart condition such as angina or atrial fibrillation; high blood pressure; kidney or liver disease; a learning disability; a mental health condition; a neurological condition such as epilepsy; a stroke (which affects your day-to-day life) and another long-term condition or disability (for this last option there was no accompanying free-text response space for the participant to specify the particular condition or disability that they were reporting).

A subsequent question, ‘Would you describe yourself as having ‘long COVID’, that is, you are still experiencing symptoms more than 12 weeks after you first had COVID-19, that are not explained by something else?’ was included in our analyses for long-term conditions.

### Patient experience measures

The survey also included a series of questions relating to participant’s experiences of primary care services, with corresponding Likert scale question response options. These question items covered five broad domains: overall experience, before trying to make an appointment, access, continuity and communication. We categorised the question responses into positive/affirmative and negative producing binary responses in line with the GPPS National Report.^17^ Question wording and categorisation of responses are outlined in online supplemental table S1.

### Missing data

The primary exposure is self-reported autistic, hence responses with missing data for long-term conditions were excluded from the analyses (9.9%). Primary comparisons are in the occurrence of long-term conditions and patient experiences of primary care. Comparisons are adjusted for age, gender, deprivation and ethnicity, thus responses with missing data for age, gender, deprivation and ethnicity were excluded from the analyses (3.9%).

### Statistical analysis

All analyses were performed by using Stata V.17. A descriptive analysis of participant demographic and socioeconomic characteristics was conducted for total respondents and stratified by whether participants self-reported as autistic or not. Unweighted frequencies are reported alongside weighted percentages (calculated using survey design and non-response weights by age, gender, geographical location and GP practice), with 95% CIs. Percentage point differences (ppd) in weighted proportions are presented with 95% CIs to enable comparisons between participants who self-reported autistic and those who did not.

To examine differences in the occurrence of long-term health conditions between participants self-reporting as autistic and those who did not, descriptive weighted percentages and 95% CIs are reported. Adjusted analyses were carried out to examine the associations between other long-term conditions and self-reporting as autistic, involving logistic regression models incorporating probability weights and adjusting for age, ethnicity, gender and area-level deprivation (IMD quintile) were fitted to return adjusted ORs (aORs) with 95% CIs and p values. Age (16–24 years, 25–34, 35–44, 45–54, 55–64, 65–74 and 75 or over), ethnicity (white, mixed or multiple ethnic groups, Asian or Asian British, black, black British, Caribbean or African and other ethnic group), gender (female, male, non-binary and prefer to self-describe) and area-level deprivation/IMD quintile (1 (most deprived); 2, 3, 4, 5 (least deprived)) were all entered into the regression model as categorical variables. Differences in the occurrence of long-term conditions between the two groups by age were investigated through the incorporation of an interaction effect between age and autism status. The marginal probability of each long-term condition according to autism status and age group was calculated and presented graphically.

Adjusted analyses (as described above) were also undertaken to compare the primary care experiences of adults who self-report as autistic with those who do not, producing weighted percentages and aORs using logistic regression. Robust SEs were used to account for variations in patient experiences that might be explained by differences within and between GP practices.

### Sensitivity analysis

We performed a sensitivity analysis which excluded patients with Alzheimer’s disease or other causes of dementia or a learning disability from the analysis of experiences of primary care, as these items may have been completed by carers and not necessarily be self-reported. As a further sensitivity analysis, we ran the analysis of experiences of primary care using different comparator groups, first comparing to those with no other long-term health conditions and second comparing to those with at least one other long-term health condition.

### Patient and public involvement

There was no patient and/or public involvement in the design, or conduct, or reporting, or dissemination plans of the analysis described in this article, though the governance of the 2022 GPPS did involve input from a steering group which included patient representatives among its members.^19^

## Results

### Frequency of self-reported autism

**Figure 1:**
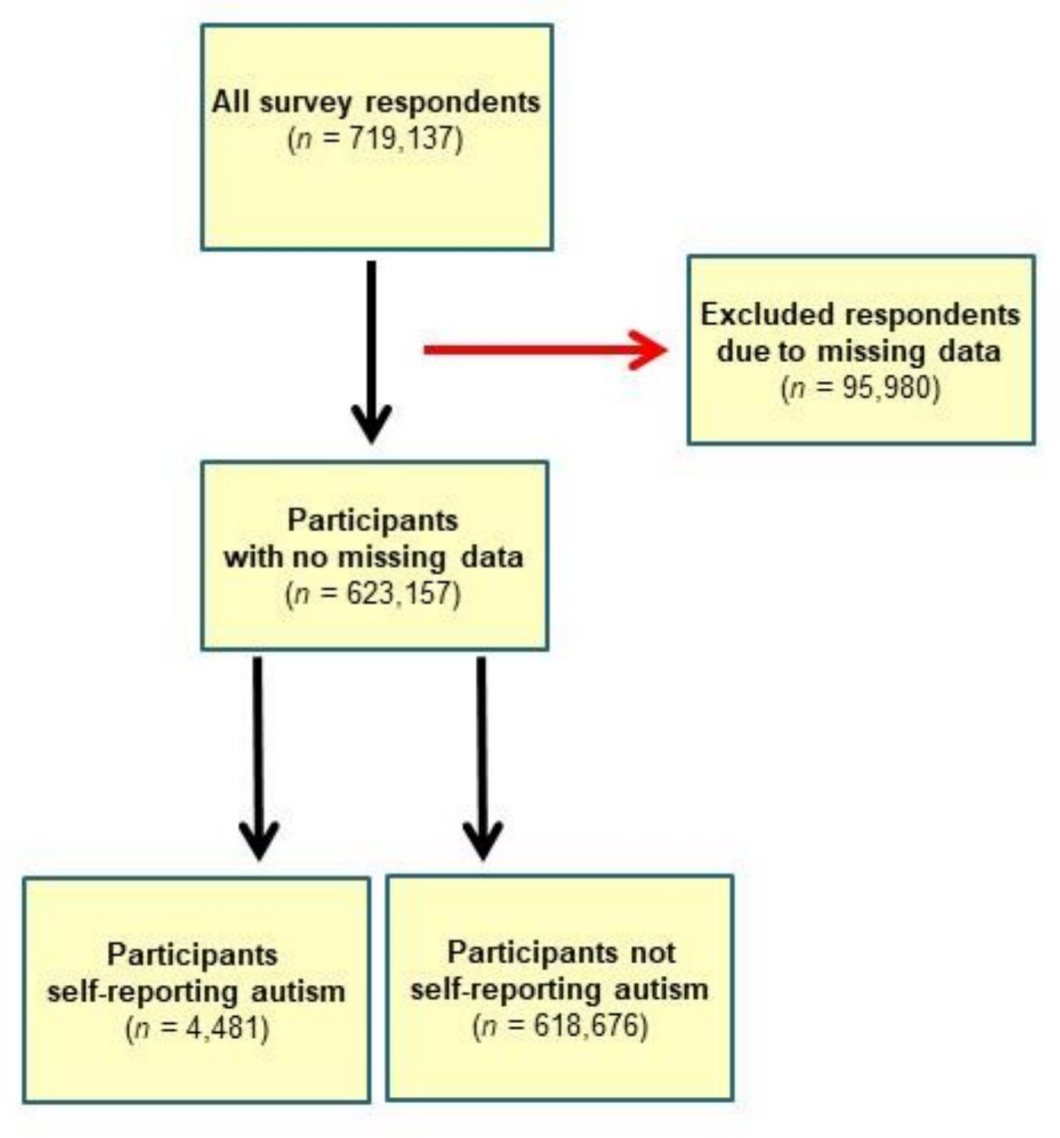
Flow chart, illustrating how the final analysis study population was attained.

Figure 1 shows a flow chart illustrating how the final analysis study population was attained. A total of 4481 of the 623 157 survey participants included in the analysis self-reported autism or an autism spectrum condition, yielding a weighted proportion estimate of 1.41% (95% CI 1.35% to 1.46%) of the sample. The 70 900 (9.9%) participants with missing data for this question were excluded from these analyses, as well as a further 25 080 (3.9%) participants with missing age, gender, ethnicity or deprivation information.

### Demographic characteristics

Table 2 summarises the demographic characteristics of all survey respondents, as well as stratified by self-reported autism status. Compared with people not self-reporting as autistic, those who self-reported as autistic were more likely to describe their gender as male (ppd+17.4%) or non-binary (ppd+3.6%) and were also more likely to describe their gender as different from the sex registered at birth (ppd+5.9%) and to describe themselves as gay or lesbian (ppd+4.3%), bisexual (ppd+8.3%) or other (ppd+5.5%). Additionally, compared with people not self-reporting as autistic, those self-reporting as autistic were younger, with higher proportions in the 16–24 years (ppd+26.7%) and 25–34 years (ppd+14.9%) age groups. They were less likely to identify as being of Asian or Asian British (ppd −4.6%) or black, black British, Caribbean or African ethnicity (ppd −1.1%) and more likely to identify with no religion (ppd+15.7%). While adults self-reporting as autistic were less likely to report having parental responsibility for a child in their household (ppd −12.8%), they were slightly more likely to report having unpaid caring responsibilities for other persons and were more likely to report undertaking these roles for 50+ hours per week than those who did not self-report as autistic. In terms of employment status, people self-reporting as autistic were less likely to be in full-time work (ppd −21.7%) and more likely to be in full-time education (ppd+12.4%), unemployed (ppd+9.6%) or permanently sick or disabled (ppd+19.7%). They were also more likely to live in the most deprived neighbourhoods (ppd+8.7% for quintile 1, associated with the greatest level of deprivation).

### Long-term conditions

Table 3 shows the reported occurrence of long-term health conditions and long COVID in people self-reporting as autistic compared with those who do not, adjusting for age, gender, deprivation and ethnicity. It should be noted that the participants self-reporting as autistic were more likely to be younger, male or non-binary, have a greater level of neighbourhood deprivation and be of white ethnicity, underlining the importance of adjusted analyses. After adjustment, all 16 conditions had higher odds of occurring among people self-reporting as autistic than those who did not. This included learning disability (aOR 18.49), mental health conditions (aOR 4.63), neurological conditions (aOR 5.03), dementia (aOR 9.20), blindness or partial sight (aOR 5.32), and deafness or hearing loss (aOR 3.02). Furthermore, following adjustment for age, gender, ethnicity and deprivation, people self-reporting as autistic had a heightened risk of having ‘another long-term condition or disability’, an additional category intended to capture conditions not recorded in the other, more specific categories.

**Table 3:** Occurrence of self-reported long-term health condition or disability, by whether self-report as autistic

Long-term health condition	Self-reported autistic (yes)N=4481			Self-reported autistic (No)N=618 676			Logistic regression*	
	N	Weighted %†	95% CI	N	Weighted %†	95% CI	aOR	95% CI
Dementia	120	1.6	(1.2, 2.0)	5128	0.6	(0.5, 0.6)	9.20	(6.96, 12.15)
Arthritis or ongoing problem with back or joints	1002	13.9	(12.7, 15.1)	150 980	17.6	(17.4, 17.7)	2.38	(2.12, 2.67)
Blindness or partial sight	216	3.7	(3.1, 4.5)	10 568	1.3	(1.3, 1.4)	5.32	(4.28, 6.61)
Breathing condition, such as asthma or COPD	857	16.1	(14.7, 17.6)	78 542	11.2	(11.1, 11.3)	1.91	(1.71, 2.13)
Cancer (diagnosis or treatment in the last 5 years)	173	2.0	(1.7, 2.5)	28 859	3.2	(3.1, 3.2)	1.97	(1.57, 2.47)
Deafness or hearing loss	403	6.2	(5.4, 7.2)	52 098	5.9	(5.8, 5.9)	3.02	(2.56, 3.56)
Diabetes	415	5.0	(4.3, 5.7)	65 654	7.8	(7.7, 7.9)	1.54	(1.32, 1.8)
Heart condition	328	4.2	(3.5, 4.9)	50 121	5.6	(5.5, 5.6)	2.19	(1.83, 2.63)
High blood pressure	591	7.3	(6.4, 8.3)	142 727	16.0	(15.9, 16.2)	1.48	(1.25, 1.75)
Kidney or liver disease	190	2.7	(2.2, 3.3)	16 127	2.0	(2.0, 2.1)	2.63	(2.14, 3.24)
Learning disability	1287	32.5	(30.5, 34.5)	5424	1.4	(1.3, 1.4)	18.49	(16.5, 20.71)
Mental health condition	2033	46.5	(44.4, 48.5)	58 866	11.8	(11.7, 12.0)	4.63	(4.23, 5.06)
Neurological condition	425	8.9	(7.9, 10)	12 538	2.0	(2.0, 2.1)	5.03	(4.36, 5.8)
Stroke (which affects your day-to-day life)	101	1.3	(1.0, 1.8)	7062	0.8	(0.8, 0.8)	4.25	(3.15, 5.73)
Other	1115	22.5	(20.9, 24.2)	90 171	13.7	(13.6, 13.8)	2.23	(2.02, 2.45)
Long COVID	291	6.7	(5.6, 7.9)	24 460	4.7	(4.7, 4.8)	1.38	(1.15, 1.67)

* Adjusted for age, gender, deprivation, and ethnicity.Weighted percentages are calculated using survey design and non-response weights by age, gender, geographic location, and GP practice.

† Weighted percentages are calculated using survey design and non-response weights by age, gender, geographical location and GP practice.

aORadjusted ORCOPDchronic obstructive pulmonary diseaseGPGeneral Practitioner

**Figure 2:**
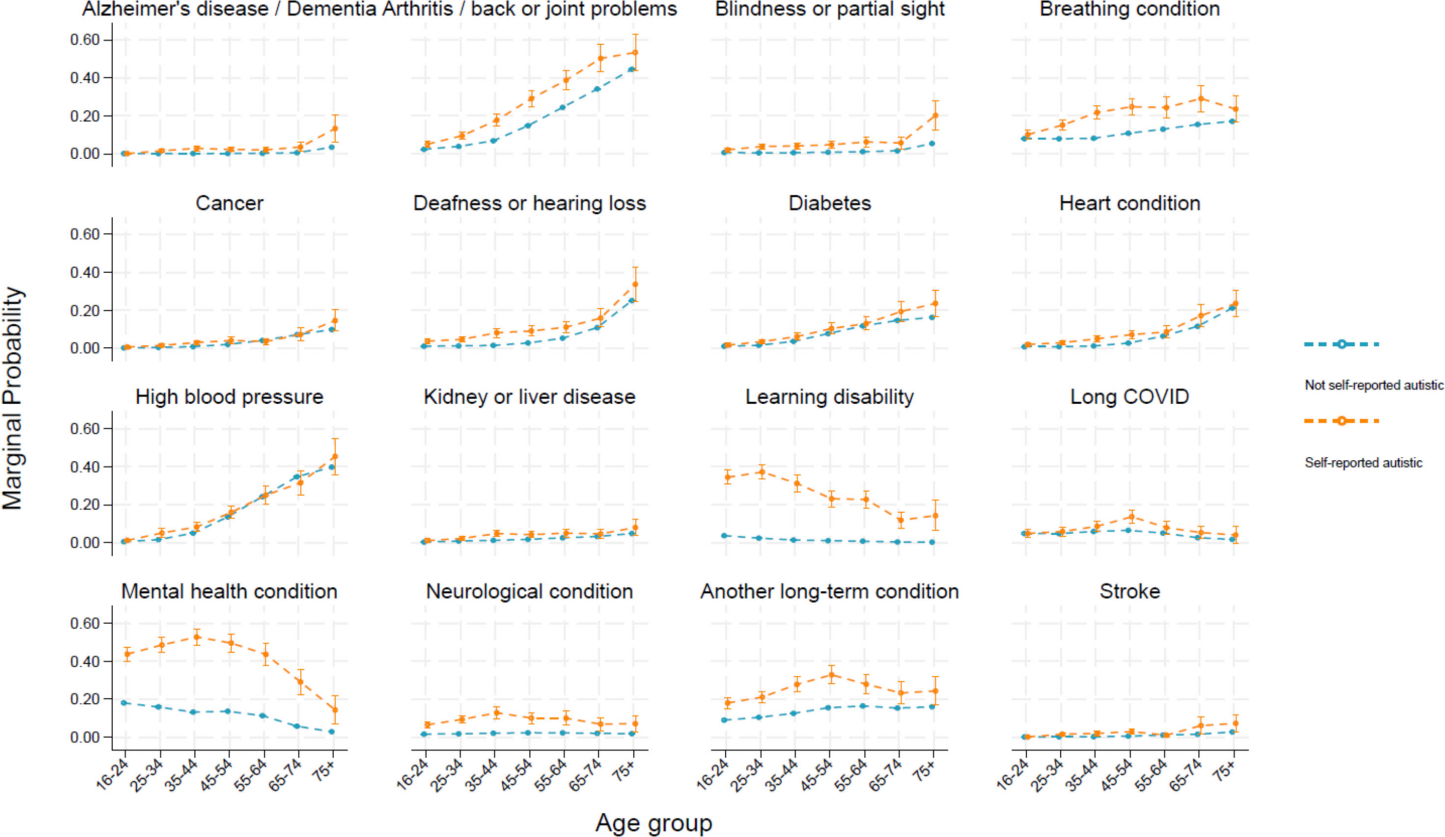
Marginal probability of long-term health condition or disability over age groups, by whether they self-report as autistic.

Figure 2 depicts differences in the marginal probability of each long-term condition between those who self-reported as autistic and those who did not by age. Conditions where there are large differences between participants with self-reported autism compared with those who do not report autism appear to be greater among younger age groups, showing some reduction in older age groups. Examples of such conditions include breathing conditions, learning disabilities, mental health conditions and neurological conditions.

### Experiences of primary care

Table 4 shows the responses to question items pertaining to patient experience. While for several question items, no significant differences were found, several distinct differences with respect to their experiences of primary care were identified.

**Table 4:** Experience of primary care, by whether self-report as autistic

	Self-reported autistic (yes)N=4481			Self-reported autistic (no)N=618 676			Logistic regression*	
	N	Weighted%†	95% CI	N	Weighted%†	95% CI	aOR	95% CI
Overall experience								
Overall positive experience of GP practice	3014	65.9	(63.9, 67.8)	472 408	72.8	(72.6, 73.0)	0.93	(0.85, 1.02)
Overall positive experience of making appointment	2228	50.8	(48.6, 52.9)	352 979	56.5	(56.3, 56.7)	0.90	(0.82, 0.98)
Before trying to make an appointment								
Used an online NHS service	843	21.5	(19.8, 23.3)	71 145	16.5	(16.4, 16.7)	0.94	(0.84, 1.04)
Used a non-NHS online service	755	19.3	(17.6, 21.0)	65 295	14.8	(14.6, 14.9)	0.98	(0.88, 1.10)
Spoke to a pharmacist	790	19.5	(17.8, 21.2)	92 301	16.4	(16.3, 16.6)	1.33	(1.19, 1.49)
Tried to treat myself	1224	29.6	(27.7, 31.6)	139 362	26.7	(26.5, 26.9)	1.05	(0.95, 1.15)
Called an NHS helpline	470	10.5	(9.3, 11.8)	38 241	8.0	(7.9, 8.1)	1.12	(0.98, 1.27)
Contacted or used another NHS service	317	7.7	(6.7, 8.9)	24 427	4.9	(4.8, 4.9)	1.38	(1.18, 1.62)
Asked for advice from friends or family	1235	35.0	(32.9, 37.1)	94 927	21.2	(21.0, 21.4)	1.25	(1.14, 1.38)
Tried to get information or advice elsewhere	624	15.6	(14.1, 17.2)	49 770	11.0	(10.9, 11.1)	1.12	(0.99, 1.26)
Access								
Easy to use GP practice’s website	1630	60.2	(57.7, 62.7)	232 330	67.3	(67.1, 67.6)	0.78	(0.70, 0.87)
Easy to get through to someone on the phone	2272	50.1	(48.0, 52.2)	349 746	52.9	(52.7, 53.1)	0.95	(0.87, 1.03)
Found the receptionists at GP practice helpful	3353	76.7	(74.8, 78.4)	508 537	82.5	(82.3, 82.6)	0.94	(0.84, 1.04)
Satisfied with GP appointment times	2070	51.6	(49.4, 53.9)	312 801	55.4	(55.2, 55.6)	1.02	(0.93, 1.12)
Satisfied with appointment offered	2478	67.7	(65.6, 69.8)	388 969	72.2	(72.0, 72.4)	0.91	(0.82, 1.00)
In-person appointment at own GP practice‡	1417	44.6	(42.3, 47.0)	224 637	46.1	(45.9, 46.3)	0.89	(0.81, 0.98)
Continuity								
Have a preferred GP	2386	54.4	(52.3, 56.5)	277 874	42.9	(42.8, 43.1)	2.25	(2.06, 2.46)
Able to see or speak to preferred GP§	939	46.4	(43.3, 49.5)	110 264	43.4	(43.1, 43.7)	1.10	(0.96, 1.25)
Communication								
Involved in decisions about care and treatment	3261	84.8	(83.2, 86.3)	470 903	90.2	(90.1, 90.3)	0.78	(0.69, 0.89)
Had mental health needs recognised and understood	2490	77.4	(75.4, 79.3)	213 142	81.1	(80.9, 81.3)	0.90	(0.80, 1.01)
Confidence and trust in healthcare professional	3543	87.6	(86.1, 88.9)	538 076	93.3	(93.2, 93.4)	0.67	(0.59, 0.77)
Needs were met	3435	84.7	(83.2, 86.1)	530 666	91.2	(91.1, 91.3)	0.73	(0.65, 0.83)

* Adjusted for age, gender, deprivation, and ethnicity.Weighted percentages are calculated using survey design and non-response weights by age, gender, geographic location, and GP practice. Base: who accepted an appointment the last time they tried to book.Base: with a preferred GP.

† Weighted percentages are calculated using survey design and non-response weights by age, gender, geographical location and GP practice.

‡ Base: Patient who accepted an appointment the last time they tried to book.

§ Base: Patients with a preferred GP.

GPGeneral PractitionerNHSNational Health Service

People self-reporting as autistic reported a less positive experience with respect to making an appointment relative to their peers (aOR 0.90). Prior to making an appointment, people self-reporting as autistic were more likely to speak to a pharmacist (aOR 1.33), contact or use another NHS service (aOR 1.38) or ask for advice from a friend or family member (aOR 1.25). However, no significant difference was reported between participant’s self-reporting as autistic compared with those not self-reporting as autistic with respect to their overall experience of their GP practice.

In relation to issues about access and continuity, people who self-reported as autistic were less likely to report their GP practice’s website as easy to use compared with their peers (aOR 0.78), as well as being less likely to be offered an in-person appointment at their own GP practice (aOR 0.89). Despite finding no differences in satisfaction with the GP appointment times available, they were less likely to be satisfied with the appointment offered (aOR 0.91). People self-reporting as autistic were more likely to have a preferred GP compared with their peers not self-reporting as autistic (aOR 2.25). No significant differences were identified with respect to being able to see or speak to their preferred GP (compared with those who did not self-report as autistic).

Regarding communication, people self-reporting as autistic were less likely to report being involved in decisions about care and treatment (aOR 0.78), having confidence and trust in their healthcare professional (aOR 0.67), or that their needs were met (aOR 0.74). However, there was no significant difference with respect to reporting having their mental health needs recognised and understood.

Findings were not substantially different after excluding patients with Alzheimer’s disease or other causes of dementia or a learning disability (online supplemental table S2). Some differences in the patient experience analyses were observed when using different comparator groups (online supplemental table S3). Relatedly, Paddison *et al*^22^ have previously analysed the impact of long-term conditions more generally, including multimorbidity, on patient primary care experiences using GPPS data.

## Discussion

### Principal findings

Adults self-reporting as autistic in England have a distinct sociodemographic profile, being more likely to report being younger, male, non-binary, have a gender different from their sex at birth, have a non-heterosexual sexual identity, be of white or mixed or multiple ethnic groups, be unemployed and live in more deprived areas. They also report higher rates of a range of long-term health conditions, including learning disability, neurological conditions, and visual and hearing impairment. While no significant difference was observed with respect to their overall experience of their GP practice, adults self-reporting as autistic reported lower confidence in healthcare professionals and were less likely to report their needs being met. Furthermore, adults self-reporting as autistic demonstrate differences with respect to help-seeking behaviours prior to making an appointment, including being more likely to report speaking to a pharmacist, contacting or using another NHS service and asking for advice from a friend or family member.

The data reported here show that people self-reporting as autistic were more likely to report taking a variety of different actions prior to attempting to make a general practice appointment (table 4). This is consistent with findings from focus groups with autistic people, where participants described feeling ‘reluctant to seek help’ and ‘that they only access primary care as a last resort.^8^’ A previous survey of autistic adults has identified barriers to visiting their GP, including being unsure if their symptoms warrant a visit, difficulty making appointments via telephone, not feeling understood, difficulty communicating with their doctor and the waiting room environment.^23^ These findings are valuable in informing public health interventions targeting this group; knowing that people self-reporting as autistic are more likely to speak to a pharmacist or seek advice from a friend or family member underlines a need to ensure public health interventions are informed by such frequently used pathways. Furthermore, there is a need to reflect as to why adults self-reporting as autistic are more likely to take these other actions prior to making a general practice appointment—for instance, is this in part a reflection of their difficulties in navigating their GP practice’s website? One goal of the NHS Long Term Plan^9^ is to improve staff understanding of the needs of autistic people, and the survey analysis reported here can help inform policy initiatives that are sensitive and responsive to the needs of the autistic community. One such approach could be specialised clinics for autistic people; evidence from the USA supports such approaches, where clinician continuity is prioritised, as well as patients’ sensory needs.^24^

The GPPS is not intended as a prevalence survey but rather aims to report on how patients feel about their GP practice, with a view to improving their healthcare. Thus, the estimated proportion of individuals self-reporting as autistic in our analysis should not be interpreted as a definitive prevalence estimate, as doing so could lead to misallocation of healthcare resources. However, for future iterations of the GPPS, it would be helpful to include additional survey question items asking participants whether a professional has assessed them for each self-reported condition (including autism), whether they have requested or are on a waiting list for an assessment, and whether they have ever had a diagnosis confirmed by a health professional. Alternatively, such evidence of the presence of a formal clinical diagnosis could be obtained through cross-checking with participants’ electronic healthcare records. This will help differentiate between patients who self-report as autistic but do not (yet) have a clinical diagnosis, and from those who have received a clinical diagnosis, and ascertain what similarities and differences exist between these groups.

### Strengths and weaknesses of the study

A major strength of this study is the sample size of 643 447 adults. The stratified random probability design and weights provide a representative sample of adults registered with GP practices across England. The focus on adults is a particular strength, as most autism epidemiology has focused on children. The self-report nature of the data is also a strength, providing insight on subjective experiences, perceptions and identities which are rarely systematically collected and stored in administrative sources.

There are limitations to the generalisability of the reported findings. While reporting an association between self-reporting as autistic with long-term conditions and primary care experiences is informative, data from the 2022 GPPS is cross-sectional in nature, and longitudinal data are required to establish causality. While high for a study of its type, the 29.1% response rate could have introduced unknown participation bias. For example, an analysis of the 2009 GPPS reported that ‘men, young adults and people living in deprived areas were under-represented among respondents,’^25^ though the weighting strategy was developed to mitigate this under-representation. Additionally, survey findings could be influenced by differential likelihood to respond by autism status, as well as whether the adults self-reporting as autistic who do respond are representative of adults self-reporting as autistic more generally. Furthermore, the survey required adults to self-report long-term conditions, including autism, and we have no means to formally confirm their responses (such as an autism diagnosis) on their medical records. However, data on people self- or proxy-reporting autism via this approach have been previously described in the research literature,^26^ and considering the high levels of autism underdiagnosis^14^ and barriers to diagnostic assessment,^27^ many adults self-reporting autism who lack a formal diagnosis may meet diagnostic criteria if they were to undergo diagnostic testing.

Evidence of the similarities between clinically diagnosed and self-reporting autistic adults comes from Sturm *et al*,^28^ using the Ritvo Autism and Asperger Diagnostic Scale-Revised (RAADS-R)^29^ and Ritvo Autism and Asperger Diagnostic Scale-Revised; RAADS-14-Item-Screen (RAADS-14)^30^ autism screening instruments, who reported ‘few psychometric differences between diagnosed and self-identifying (autistic) individuals.’ Additionally, McDonald^31^ found that in using the Autism Spectrum Identity Scale,^31^ a measure of autism identity, both diagnosed and self-identifying autistic adults provided very similar results with respect to stigma, self-esteem, quality of life and autism identity. Thus, while a level of bias may have been introduced through the self-reporting nature of autism in the context of the GPPS, research evidence suggests that those who self-identify as autistic without an accompanying diagnosis share important similarities to those with a formal diagnosis. Considering the barriers to obtaining an autism diagnosis, high levels of underdiagnosis,^14^ and the similarities that exist between these two groups, a large sample of patients self-reporting as autistic may provide a more accurate reflection of autistic adults in England compared with focussing solely on those with a formal diagnosis.

It is also possible that some adults self-reporting as autistic may have received support from a carer in undertaking the survey, particularly those with co-occurring learning disability, which may have impacted on the responses provided. However, no data were collected with respect to whether any surveys were completed by a proxy. Additionally, an analysis of the 2015–16 GPPS reported that respondents were more likely to report having had a GP appointment in the past year when compared with other surveys of healthcare utilisation.^32^ This could potentially lead to the GPPS overestimating the frequency of long-term conditions, though an analysis of the 2011–2012 survey conducted by Mujica-Mota *et al*^33^ reported that the GPPS long-term condition estimates were broadly comparable with those reported from other surveys, with the exception of diabetes, high blood pressure and back problems, where GPPS estimates were approximately 33%–60% greater. The study sample does not include those not registered with a GP, nor people living in residential settings, though this group comprises a small proportion of the general population. Furthermore, the GPPS only covers England, so the findings reported here may not be generalisable to other nations.

### Strengths and weaknesses in relation to other studies

The findings reported here should be compared with those from the Adult Psychiatric Morbidity Survey (APMS), a national survey of mental health and well-being among community-based adults in England.^34^ Participants were identified with autism according to assessment with the Autism Diagnostic Observation Schedule,^35^ undertaken by clinically trained interviewers, to ensure that the autism identification process was broadly similar to that used in clinical practice.^36^ The estimated proportion of adults identified with autism from the combined 2007 and 2014 samples was around 0.8% (95% CI 0.5% to 1.3%),^36^ slightly lower than the 1.41% (95% CI 1.35% to 1.46%) reported here. However, this estimate increased to 1.1% (95% CI 0.3% to 1.9%) when accounting for adults with intellectual disability, who were not included in the APMS,^37^ though are in the GPPS; thus, this latter estimate likely represents a more accurate figure when comparing with the GPPS respondent population. Furthermore, the sample identified with autism in the APMS did not demonstrate a clear pattern in age distribution, or employment status, in contrast to the GPPS findings reported here.

With respect to autistic adults having an elevated burden of long-term conditions, our findings are similar to those previously reported.^38 39^ However, we report findings for adults self-reporting as autistic, rather than only those who have confirmed diagnoses on their healthcare records.^38 39^ Additionally, while attempts have previously been made to evaluate the healthcare experiences of autistic adults in different contexts, such as regarding their mental health,^40^ to the author’s knowledge, this is the first analysis of a national survey of autistic adults experiences relating to primary care.

### Conclusions

The proportion of general practice registered adults self-reporting as autistic is similar to previously reported autism estimates using APMS data in combination with data from adults with intellectual disability.^37^ Adults self-reporting as autistic have a distinct sociodemographic profile and are more likely to report having a wide range of long-term conditions. They are also more likely to report challenges with respect to accessing primary care and having their needs met when they do, which is in line with the APMS population survey results. Such findings can be relied on therefore to inform approaches to improving the healthcare experiences of adults self-reporting as autistic within primary care settings.

## supplementary material

10.1136/bmjopen-2023-081388 online supplemental file 1

## Data Availability

Data may be obtained from a third party and are not publicly available.
