# Supplementary material for: Characteristics and primary care experiences of people who self-report as autistic: a probability sample survey of adults registered with primary care services in England
Source: BMJ Open. 2024 Sep 13;14(9):e081388. doi: 10.1136/bmjopen-2023-081388 (PMC11404134; doi:10.1136/bmjopen-2023-081388)
Supplement: online supplemental file 1 [file bmjopen-14-9-s001.pdf]

Supplementary Tables

Table S1: Patient experience questions: wording and categorisation of responses

	Question	Positive/affirmative responses	Negative responses	Exclusions
**Overall experience**				
Overall positive experience of GP practice	Overall, how would you describe your experience of your GP practice?	'Very good'<br>'Fairly good'	'Neither good nor poor'<br>'Fairly poor'<br>'Very poor'	
Overall positive experience of making appointment	Overall, how would you describe your experience of making an appointment?	'Very good'<br>'Fairly good'	'Neither good nor poor'<br>'Fairly poor'<br>'Very poor'	
**Before trying to make an appointment**	Before you tried to get this appointment, did you do any of the following?			
Used an online NHS service		Used an online NHS service (including NHS 111 online)		
Used a non-NHS online service		Used a non-NHS online service, or looked online for information		
Spoke to a pharmacist		Spoke to a pharmacist		
Tried to treat myself		Tried to treat myself / the person I was making this appointment for (for example with medication)		
Called an NHS helpline		Called an NHS helpline, such as NHS 111		
Contacted or used another NHS service		Contacted or used another NHS service		
Asked for advice from friends or family		Asked for advice from a friend or family member		
Tried to get information or advice elsewhere		Tried to get information or advice elsewhere (from a non-NHS service)		
**Access**				
Easy to use GP practice's website	How easy is it to use your GP practice's website to look for information or access services?	'Very easy'<br>'Fairly easy'	'Not very easy'<br>'Not at all easy'	'Haven't tried'
Easy to get through to someone on the phone	Generally, how easy is it to get through to someone at your GP practice on the phone?	'Very easy'<br>'Fairly easy'	'Not very easy'<br>'Not at all easy''	'Haven't tried'
Found the receptionists at GP practice helpful	How helpful do you find the receptionists at your GP practice?	'Very helpful'<br>'Fairly helpful'	'Not very helpful'<br>'Not at all helpful'	'Don't know'
Satisfied with GP appointment times	How satisfied are you with the general practice appointment times that are available to you?	'Very satisfied'<br>'Fairly satisfied'	'Neither satisfied nor dissatisfied'<br>'Fairly dissatisfied'<br>'Very dissatisfied'	'I'm not sure when I can get an appointment

Satisfied with appointment offered	Were you satisfied with the appointment (or appointments) you were offered?	‘Yes, and I accepted an appointment’	‘No, but I still took an appointment’<br>‘No, and I did not take an appointment’	‘I was not offered an appointment’
In-person appointment at own GP practice[1]	What type of appointment did you get?	‘…to see someone ay my GP practice’	‘…to speak to someone on the phone’<br>‘…to see someone at another general practice location’<br>‘…to speak to someone online (for example on a video call)’<br>‘…for a home visit’	
**Continuity**				
Have a preferred GP	Is there a particular GP you usually prefer to see or speak to?	‘Yes, for all appointments’<br>‘Yes, for some appointments but not others’	‘No’	‘There is usually only one GP in my GP practice’
Able to see or speak to preferred GP[2]	How often do you see or speak to your preferred GP when you would like to?	‘Always or almost always’<br>‘A lot of the time’	‘Some of the time’<br>‘Never or almost never’	‘I have not tried’
**Communication**				
Involved in decisions about care and treatment	During your last general practice appointment, were you involved as much as you wanted to be in decisions about your care and treatment?	‘Yes, definitely’<br>‘Yes, to some extent’	‘No, not at all’	‘Don’t know / doesn’t apply’
Had mental health needs recognised and understood	During your last general practice appointment, did you feel that the healthcare professional recognised and/or understood any mental health needs that you might have had?	‘Yes, definitely’<br>‘Yes, to some extent’	‘No, not at all’	‘I did not have any mental health needs’<br>‘Did not apply to my last appointment’
Confidence and trust in healthcare professional	During your last general practice appointment, did you have confidence and trust in the healthcare professional you saw or spoke to?	‘Yes, definitely’<br>‘Yes, to some extent’	‘No, not at all’	‘Don’t know / doesn’t apply’
Needs were met	Thinking about the reason for your last general practice appointment, were your needs met?	‘Yes, definitely’<br>‘Yes, to some extent’	‘No, not at all’	‘Don’t know / can’t say’

[1]Base: Patient who accepted an appointment the last time they tried to book (To the question: were you satisfied with the appointment (or appointments) you were offered? Responded ‘Yes, and I accepted an appointment’ or ‘No, but I still took an appointment’).
[2]Base: Patients with a preferred GP (To the question: is there a particular GP you usually prefer to see or speak to? Responded ‘Yes, for all appointments’, or ‘Yes, for some appointments but not others’).

Table S2: Experience of primary care, by whether self-report as autistic: sensitivity analysis excluding patients with Alzheimer's disease or other cause of dementia or a learning disability (excluding n=11,742 patients; 1.9%).

	Self-reported autistic (Yes) N=3,143		Self-reported autistic (No) N=608,272		Logistic regression[1]	
	Weighted %[2]	95% CI	Weighted %[2]	95% CI	aOR	95% CI
**Overall experience**						
Overall positive experience of GP practice	64.3	(61.9, 66.6)	72.8	(72.6, 73.0)	0.85	(0.77, 0.95)
Overall positive experience of making appointment	49.2	(46.7, 51.7)	56.5	(56.3, 56.7)	0.84	(0.76, 0.93)
**Before trying to make an appointment**						
Used an online NHS service	24.2	(22.1, 26.5)	16.5	(16.3, 16.7)	1.11	(0.98, 1.26)
Used a non-NHS online service	21.6	(19.5, 23.9)	14.9	(14.7, 15.0)	1.14	(1.00, 1.30)
Spoke to a pharmacist	17.8	(16.0, 19.8)	16.4	(16.3, 16.5)	1.20	(1.05, 1.37)
Tried to treat myself	30.5	(28.2, 33.0)	26.8	(26.6, 27.0)	1.09	(0.97, 1.22)
Called an NHS helpline	10.7	(9.3, 12.3)	7.9	(7.8, 8.1)	1.17	(1.00, 1.38)
Contacted or used another NHS service	7.8	(6.5, 9.2)	4.8	(4.7, 4.9)	1.42	(1.17, 1.71)
Asked for advice from friends or family	35.2	(32.8, 37.8)	21.1	(21.0, 21.3)	1.29	(1.15, 1.45)
Tried to get information or advice elsewhere	17.5	(15.6, 19.6)	11.1	(10.9, 11.2)	1.29	(1.12, 1.49)
**Access**						
Easy to use GP practice's website	59.6	(56.6, 62.5)	67.5	(67.2, 67.7)	0.75	(0.66, 0.85)
Easy to get through to someone on the phone	48.2	(45.7, 50.7)	52.9	(52.7, 53.1)	0.87	(0.79, 0.97)
Found the receptionists at GP practice helpful	75.3	(73.0, 77.4)	82.5	(82.3, 82.6)	0.85	(0.75, 0.96)
Satisfied with GP appointment times	48.7	(46.1, 51.4)	55.3	(55.1, 55.5)	0.91	(0.82, 1.02)
Satisfied with appointment offered	65.5	(62.9, 68.1)	72.1	(72.0, 72.3)	0.82	(0.73, 0.92)
In-person appointment at own GP practice[3]	43.6	(40.9, 46.5)	46.1	(45.9, 46.3)	0.86	(0.77, 0.97)
**Continuity**						
Have a preferred GP	52.4	(49.9, 54.9)	42.7	(42.5, 42.9)	2.08	(1.88, 2.31)
Able to see or speak to preferred GP[4]	46.9	(43.3, 50.6)	43.3	(43.1, 43.6)	1.11	(0.95, 1.29)
**Communication**						
Involved in decisions about care and treatment	84.1	(82.0, 85.9)	90.2	(90.1, 90.3)	0.73	(0.63, 0.84)
Had mental health needs recognised and understood	75.9	(73.3, 78.2)	81.1	(80.8, 81.3)	0.81	(0.71, 0.93)
Confidence and trust in healthcare professional	86.6	(84.8, 88.3)	93.4	(93.3, 93.5)	0.60	(0.52, 0.71)
Needs were met	84.1	(82.1, 85.8)	91.3	(91.1, 91.4)	0.68	(0.59, 0.79)

[1]Adjusted for age, gender, deprivation, and ethnicity

[2]Weighted percentages are calculated using survey design and non-response weights by age, gender, geographic location, and GP practice.

[3]Base: Patient who accepted an appointment the last time they tried to book.

[4]Base: Patients with a preferred GP.

Table S3: Experience of primary care, by whether self-report as autistic: sensitivity analysis using different comparator groups: (1) those with no other long-term health conditions and (2) those with at least one other long-term health conditions.

	Self-reported autistic (Yes) N=4,481		Comparator group 1: No other long-term conditions N=211,995		Comparator group 2: Another long-term condition N=406,681		Logistic regression (Comparator group 1)[1]		Logistic regression (Comparator group 2)[1]	
	Weighted %[2]	95% CI	Weighted %[2]	95% CI	Weighted %[2]	95% CI	aOR	95% CI	aOR	95% CI
**Overall experience**										
Overall positive experience of GP practice	72.2	(68.1, 75.9)	72.9	(72.6, 73.1)	72.8	(72.5, 73.0)	1.19	(0.98, 1.46)	0.96	(0.87, 1.06)
Overall positive experience of making appointment	55.7	(51.0, 60.3)	58.4	(58.1, 58.7)	55.2	(54.9, 55.4)	0.99	(0.82, 1.20)	0.97	(0.88, 1.07)
**Before trying to make an appointment**										
Used an online NHS service	21.4	(17.7, 25.8)	18.6	(18.3, 18.9)	15.0	(14.9, 15.2)	0.92	(0.72, 1.18)	0.88	(0.78, 0.99)
Used a non-NHS online service	13.9	(10.9, 17.6)	16.6	(16.3, 16.8)	13.5	(13.3, 13.7)	0.67	(0.51, 0.89)	1.00	(0.88, 1.14)
Spoke to a pharmacist	16.7	(13.4, 20.6)	15.9	(15.6, 16.1)	16.9	(16.7, 17.0)	1.22	(0.94, 1.58)	1.29	(1.14, 1.46)
Tried to treat myself	20.7	(17.3, 24.7)	26.4	(26.2, 26.7)	26.9	(26.7, 27.1)	0.73	(0.58, 0.92)	1.02	(0.92, 1.14)
Called an NHS helpline	9.8	(7.2, 13.3)	8.2	(8.0, 8.4)	7.9	(7.7, 8.0)	1.09	(0.77, 1.53)	1.04	(0.90, 1.20)
Contacted or used another NHS service	4.0	(2.4, 6.7)	4.5	(4.4, 4.6)	5.1	(5.0, 5.2)	0.77	(0.45, 1.32)	1.36	(1.15, 1.61)
Asked for advice from friends or family	30.1	(25.9, 34.7)	23.7	(23.4, 24.0)	19.4	(19.2, 19.6)	0.95	(0.77, 1.18)	1.26	(1.13, 1.41)
Tried to get information or advice elsewhere	11.8	(9.1, 15.1)	11.9	(11.7, 12.1)	10.4	(10.2, 10.5)	0.87	(0.65, 1.16)	1.08	(0.94, 1.23)
**Access**										
Easy to use GP practice's website	67.2	(61.5, 72.5)	68.8	(68.4, 69.1)	66.3	(66.0, 66.6)	0.92	(0.72, 1.18)	0.83	(0.74, 0.94)
Easy to get through to someone on the phone	54.9	(50.2, 59.6)	54.9	(54.6, 55.2)	51.5	(51.2, 51.7)	1.01	(0.84, 1.23)	1.03	(0.93, 1.13)
Found the receptionists at GP practice helpful	83.4	(79.7, 86.5)	82.2	(81.9, 82.4)	82.7	(82.5, 82.9)	1.32	(1.02, 1.69)	0.97	(0.87, 1.09)

Satisfied with GP appointment times	55.2	(50.4, 59.9)	55.4	(55.1, 55.8)	55.3	(55.1, 55.6)	1.13	(0.93, 1.38)	1.07	(0.97, 1.19)
Satisfied with appointment offered	73.6	(68.8, 77.8)	73.7	(73.4, 74.0)	71.1	(70.9, 71.3)	1.04	(0.82, 1.32)	1.00	(0.89, 1.11)
In-person appointment at own GP practice[3]	49.0	(43.8, 54.1)	49.9	(49.6, 50.3)	43.3	(43.1, 43.6)	0.85	(0.69, 1.05)	1.07	(0.95, 1.19)
**Continuity**										
Have a preferred GP	43.3	(39.0, 47.8)	33.1	(32.8, 33.4)	50.3	(50.1, 50.6)	2.11	(1.76, 2.54)	1.77	(1.60, 1.96)
Able to see or speak to preferred GP[4]	41.8	(34.9, 49.0)	40.7	(40.2, 41.3)	44.7	(44.4, 45.1)	0.99	(0.74, 1.33)	1.06	(0.92, 1.22)
**Communication**										
Involved in decisions about care and treatment	88.3	(84.4, 91.3)	90.8	(90.6, 91.0)	89.8	(89.6, 89.9)	0.90	(0.65, 1.26)	0.87	(0.76, 0.99)
Had mental health needs recognised and understood	81.8	(77.3, 85.7)	82.6	(82.2, 83.0)	80.2	(79.9, 80.5)	0.98	(0.74, 1.30)	0.97	(0.85, 1.10)
Confidence and trust in healthcare professional	90.4	(87.1, 93.0)	94.0	(93.8, 94.1)	92.9	(92.7, 93.0)	0.70	(0.50, 0.98)	0.80	(0.69, 0.93)
Needs were met	88.4	(84.8, 91.2)	91.7	(91.5, 91.9)	90.9	(90.8, 91.1)	0.84	(0.62, 1.15)	0.83	(0.73, 0.95)

[1]Adjusted for age, gender, deprivation, and ethnicity

[2]Weighted percentages are calculated using survey design and non-response weights by age, gender, geographic location, and GP practice.

[3]Base: Patient who accepted an appointment the last time they tried to book.

[4]Base: Patients with a preferred GP.
