# Supplementary material for: Characteristics and primary care experiences of people who self-report as autistic: a probability sample survey of adults registered with primary care services in England
Source: BMJ Open. 2024 Sep 13;14(9):e081388. doi: 10.1136/bmjopen-2023-081388 (PMC11404134; doi:10.1136/bmjopen-2023-081388)
Supplement: Supplementary file 2 [file bmjopen-2023-081388.reviewer_comments.pdf]

## PEER REVIEW HISTORY

BMJ Open publishes all reviews undertaken for accepted manuscripts. Reviewers are asked to complete a checklist review form and are provided with free text boxes to elaborate on their assessment. These free text comments are reproduced below.

## ARTICLE DETAILS

| | |
|---|---|
| **TITLE (PROVISIONAL)** | The characteristics and primary care experiences of people who self-report as autistic: a probability sample survey of adults registered with primary care services in England |
| **AUTHORS** | Tromans, Samuel; Teece, Lucy; Saunders, Catherine; McManus, Sally; Brugha, Traolach |

## VERSION 1 - REVIEW

| | |
|---|---|
| **REVIEWER NAME** | ***Hull, Laura*** |
| **REVIEWER AFFILIATION** | University College London |
| **REVIEWER CONFLICT OF INTEREST** | Na |
| **DATE REVIEW RETURNED** | 05-Jan-2024 |

| | |
|---|---|
| **GENERAL COMMENTS** | This study utilises a large, representative dataset to compare the characteristics and primary healthcare experiences of adults in the UK who self-report as autistic, compared to those who do not. It offers useful insights into a population who may not always be included in clinical studies, although its implications are somewhat limited by lack of information on participants with or without a formal diagnosis of autism. The focus of the study seems split into two discrete areas – patient characteristics and experiences accessing primary healthcare – which could be better connected, although I believe both areas are important. The findings have implications for changes to primary healthcare practice, although results related to characteristics could be more fully compared to existing clinical sample prevalence and characteristic data.<br>Comments:<br>Abstract – Results: I assume that male and non-binary were discrete options, in which case I'd suggest rephrasing this to 'male or non-binary'.<br>Introduction – I found the justification for this study a little hard to follow, as it feels like two different studies (characteristics/co-morbidities, and experiences of primary care) combined into one. The authors could make clearer the need for information about primary care experiences within this population.<br>Methods – it's not clear to me if there was any way to determine whether participants had a formal diagnosis of autism (whether or not they self-identify). To me, this is a limitation of the study as we don't know which participants had a formal diagnosis versus those who did not, which would be particularly novel and clinically relevant. As participant-level data was reported, could medical records be screened to identify those with a formal diagnosis? This seems particularly relevant as Table 1 implies that you might be able to distinguish between sub-types within each participant group.<br>I notice that one of the long-term health condition options was "another condition". Did the authors screen these responses to identify any relevant responses? I wonder if, in particular, self- |

| | |
|---|---|
| | identified autistic people might write something to the effect that they self-identify in this section, or might provide more context to their answer.<br>Results<br>Page 10 – when reporting the demographic characteristics please clarify what the comparison group is; this is sometimes unclear (i.e. that people self-reporting as autistic were more likely to be male than those not reporting as autistic, rather than that this subgroup was more likely to be male than other gender identities?).<br>Please add the ppd to the results reported for living in deprived neighbourhoods, for consistency (page 10, line 54).<br>Page 11 – The sentence starting “Conditions where there are large differences…” (lines 23-30) is quite long and hard to follow. I’d suggest rephrasing and maybe breaking up into two sentences to give the examples and also make the point about there being greater differences between groups in younger people.<br>The sensitivity analysis results reported in Table S3 (between different comparator groups) are interesting; I would like to see more discussion of these results although I appreciate the word count limits. In particular, these findings suggest some areas in which GP practices could make changes to improve the experiences of people with long-term conditions generally, which would also benefit autistic people, as well as areas in which autistic people may need specific adjustments or support.<br>Discussion<br>I would like some more discussion about whether the characteristics findings are similar to those of other population-based studies in the UK which categorise participants based on clinical diagnosis. If the findings are broadly similar, it might suggest that using self-reported autism as an identifying category in research would allow greater inclusion of participants who might otherwise be excluded – the undiagnosed individuals identified in the study by O’Nions et al that the authors reference.<br>A key limitation that I think needs to be acknowledged is the lack of specificity in terms of participants being ‘people who self-report as autistic’ – i.e. not knowing whether these individuals are actually autistic (which I recognise is a subjective and contentious statement!). The authors note in Table 1 that people who self-report may not actually meet diagnostic criteria, and that there will be people who do not self-report who would meet diagnostic criteria, but I think there needs to be more consideration of how large these sub-groups could be. Overall I was left uncertain of whether or not the authors felt this sample represented a more accurate assessment of autistic people in the UK than a sample comprised of clinically diagnosed people. There could be some more discussion of why people choose to self-identify as autistic and whether they have similar characteristics to those who receive a formal diagnosis, perhaps in the introduction. I’m aware there is limited space, but this is a complicated issue and I think unfamiliar readers may need some more understanding of the context. |

| | |
|---|---|
| **REVIEWER NAME** | ***Oakley, Bethany*** |
| **REVIEWER AFFILIATION** | King's College London |
| **REVIEWER CONFLICT OF INTEREST** | Na |
| **DATE REVIEW RETURNED** | 18-Mar-2024 |

| GENERAL COMMENTS | The authors utilise a very large sample drawn from a cross-sectional survey of adults registered with NHS GP surgeries in England, and demonstrate that there were some key differences in demographic features (e.g., higher rates of unemployment, higher rates of non-binary identity/differential gender identity to sex at birth) and health and social care experiences (e.g., higher rates of physical/mental health problems, reduced satisfaction in experiences interacting with services) between those who self-reported autism as a long-term health condition, as compared to those who did not in the survey. The overarching findings with regards to the experiences of autistic adults in England are important.<br>I had a couple of more minor specific points (please see below), however my main issue with the manuscript as it is currently framed is that the authors seem to place a big emphasis on the self reporting of autism here, when it is not clear that they were able to differentiate within this between those who self reported autism because they had received a formal diagnosis and those who self reported autism but had never received a formal diagnosis. This is noteworthy for two key reasons. Firstly, the authors imply in the summary box that what this study adds is evidence of experience of those self-reporting as autistic (beyond formal diagnosis), when it seems currently that the proportion of those within the 4,481 respondents who do not have a formal diagnosis is unknown. Therefore, the specific added value of this study needs to be reframed and/or clarified further (e.g., possibly this is more around the experiences of autistic adults, as compared to non autistic adults and those with other long term health conditions). Second, there is an increasing number of studies that do include self-identified autistic people without a formal diagnosis in research (for many of the reasons pointed out by the authors in their Introduction) and ongoing debates around whether or not this group are comparable to those who are formally diagnosed (e.g., there may be an over-representation of females in the self identified group, for instance) – the current framing of the paper feels a bit misleading, given this growth in research interest in self identification/diagnosis of autism.<br>Introduction<br>In the summary box, one of the bullet points is “Autism is underdiagnosed in adults.”, but I would feel a statement such as “Late diagnosis of autism (i.e., in adulthood) indicates that there is likely a notable group of adults who are undiagnosed.” [or the statement from the Introduction: “Adult general population surveys suggest that most adults with autism are not recognised (undiagnosed).”] Would be more accurate.<br>In the Introduction where the authors point out “In addition, many people may consider themselves to be autistic but who would not meet diagnostic thresholds if for autism assessed although they may have other significant, unidentified mental health conditions.” – it might be informative to add some detail around some of the underlying reasons for this (i.e., because those individuals are more likely to start off in mental health services, as is commonly reported in later diagnosed autistic females, for instance).<br><br>Method<br>In the section ‘self reported autism definition’, it’s not clear to me how the item option ‘Autism or autism spectrum condition’ to the multiple choice question ‘Which, if any, of the following long-term conditions do you have?’ could differentiate between adults with self-reported autism and adults who did not self report autism as per the total definition provided by the authors in Table 1. In other words, what was the specific question that ascertained self |
|---|---|

| | |
|---|---|
| | identification of autism (where there was no clinical diagnosis)?<br>In addition, were the authors able to also differentiate within the ‘not self reporting as autistic’ group between those who did have a clinical diagnosis, and those who did not (i.e., to compare those who self identify as autistic but do not have a clinical diagnosis of autism both to those who have a clinical diagnosis of autism, and to a general population sample of those who neither self identify as autistic nor have a clinical diagnosis of autism)?<br><br>Results/Discussion<br>The Results are interesting and stark, however the Discussion would benefit from greater emphasis on the interpretation and implications of the specific findings, as integrated more closely into the existing broader literature. There seemed to be a greater focus here on strengths/limitations of the work (though this did not touch upon the issue outlined above regarding the lack of clarity over who is included in the self reporting as autistic group’). Particularly, expansion on clinical/policy implications to address the health and social care experiences reported by autistic adults would be critical. |

| | |
|---|---|
| **REVIEWER NAME** | ***Campbell, John*** |
| **REVIEWER AFFILIATION** | University of Exeter, Primary Care |
| **REVIEWER CONFLICT OF INTEREST** | Have previously worked with and published with corresponding author. But >8 years ago, and limited professional relationship at time. I am one of original designers of main survey isntrument used (GPPS) in thsi study. |
| **DATE REVIEW RETURNED** | 24-Apr-2024 |

| | |
|---|---|
| **GENERAL COMMENTS** | Thank you for the opportunity to review and comment on the report of this interesting study examining the characteristics and primary care experiences of people who self-report at autistic.<br><br>The work is of relevance since it reports research on an underserved UK population. The research itself is based on GP Patient Survey data – this being a large routine survey undertaken and overseen by the NHS. In general, I found the work to be excellent – highly relevant and potentially important identifying gaps in the primary care system – particularly in respect of this vulnerable population.<br><br>The authors draw on GPPS data to identify the characteristics of individuals self-reporting as autistic, comparing this with other survey respondents. Several distinctive characteristics are identified.<br><br>My main concern with the study relates to table 3 which I find confusing. The table presents a number of comparisons for individuals who self-reported as autistic as compared to those who did not, presenting findings as adjusted odds ratios. However, those reporting as autistic sometimes had increased or decreased prevalence of long-term health conditions but the adjusted odds ratios are always positive and >1 despite the reduced prevalence - for example in respect of diabetes or heart conditions. I presume this has to do with the “adjustments” being made in the analysis but in the absence of univariate associations it is difficult to understand this somewhat surprising finding. This may be my lack of understanding and statistical expertise, but surely one would expect the adjusted odds ratio to be less <1 where the prevalence was reduced against the comparator group. |

| | |
|---|---|
| | The authors do consider the issue of self-reporting against confirmed diagnosis (in 7/47 etc.) but I would like to see some more detail on the implications of self-reporting and this reporting bias in respect of the results presented.<br><br>Data for GPPS 2002 was used to undertake this study – under what circumstances and arrangements was this data made available to the study team?<br><br>Is 9/48 correct in terms of grammar/presentation<br><br>10/40 is this about the marginal probability of reporting of each long-term condition – is something missing in this sentience?<br><br>10/58 What is the potential for completion of the questionnaire by carers on behalf of autistic respondents?<br><br>11/21 – typo “conditions”<br><br>12/34-38 notes that, for several questions, no significant differences were found between items. The authors then go on to focus on areas where substantial differences were observed. However, overall experience of GP care is a key national metric which I think should be specifically noted – there were no difference between autistic and non-autistic participants as per table 4.<br><br>13/3 – typo<br><br>15/8 – typo<br><br>Overall, I thought this was an excellent and well-presented piece of work but with some important issues, particularly in respect of table 3. |

| | |
|---|---|
| **REVIEWER NAME** | ***Kurth, Tobias*** |
| **REVIEWER AFFILIATION** | Charité Universitätsmedizin Berlin, Institute of Public Health |
| **REVIEWER CONFLICT OF INTEREST** | TK has received research grants from the German Federal Joint Committee (G-BA) and the Federal Ministry of Health (BMG). He also received personal compensation from Eli Lilly & Company, Novartis, the BMJ, and Frontiers. |
| **DATE REVIEW RETURNED** | 16-May-2024 |

| | |
|---|---|
| **GENERAL COMMENTS** | In this population-based, cross-sectional study, Tromans and colleagues aimed to identify the characteristics and primary care experiences of people who self-report as autistic. Of 623,157 responders to the survey, 4,481 self-reported as autistic. Those self-reporting as autistic were more likely to be younger, male, non-binary, to identify as a gender different from their sex at birth, have a non-heterosexual sexual identity, be of White or mixed or multiple ethnic groups, non-religious, without caring responsibilities, unemployed, live in more deprived areas, and not smoke. In an adjusted multivariable logistic regression model, the authors further investigated chronic conditions that were more prevalent among adults self-reporting as autistic.<br>This is an important study covering an overlooked population. Part of the results of the study will be useful to better understand the |

| | |
|---|---|
| | primary care needs and experiences of people self-reporting as autistic. However, other aspects of the study are not interpretable. The authors may want to consider the following comments.<br><br>1. The aim of the study is clearly descriptive, and the first part of the study (i.e., Table 2) uses methods to describe the characteristic patterns of people self-reporting as autistic. However, the rationale of the adjusted logistic regression model is not clear. An adjusted model aims to address potential confounding in the causal research domain, which, however, was not the aim of the study. How can the adjusted odds ratios be interpreted? What does it mean to show effect estimates in a hypothetical world where participants have the same age, gender, deprivation, and ethnicity? Why adjust for these factors? Further, adjusting for some of these factors, particularly ethnicity, may result in structural biases (http://dx.doi.org/10.1093/epirev/mxad010). The authors should be clear about the research question that is associated with this part of the study. I recommend staying in the descriptive domain and use, for example, pattern recognition or bootstrap techniques to describe the ranking or importance of certain variables. Alternatively, univariate odds ratios can be shown.<br><br>2. Part of the conclusions of the authors, in particular when calling for public health interventions, require a proper causal framework. Such conclusions are, therefore, not supported by the provided data.<br><br>3. The authors should be more specific on how age, ethnicity, gender, and deprivation score were used in the logistic regression model.<br><br>4. I may have missed it, but did the study have ethical approval?<br><br>5. The authors mention two people from Ipsos for their support. What was their support, and how was Ipsos involved in the study?<br><br>6. I may have missed it, but what statistical software was used to analyze the data?<br><br>7. As the authors report P values (which is unnecessary), they should specify the alpha level and whether the reported P values are one or two-tailed.<br><br>8. I suggest removing the number of people who self-reported as autistic from the study's title.<br><br>9. I suggest removing the statement that this is the largest study on this topic from the What This Study Adds section.<br><br>10. A flow chart will help readers to understand the final study sample. |

**VERSION 1 – AUTHOR RESPONSE**

**REVIEWER ONE**

**Comment #1**

***Comment***

**This study utilises a large, representative dataset to compare the characteristics and primary healthcare experiences of adults in the UK who self-report as autistic, compared to those who do not. It offers useful insights into a population who may not always be included in clinical studies, although its implications are somewhat limited by lack of information on participants with or without a formal diagnosis of autism. The focus of the study seems split into two discrete areas – patient characteristics and experiences accessing primary healthcare – which could be better connected, although I believe both areas are important. The findings have implications for changes to primary healthcare practice, although results related to characteristics could be more fully compared to existing clinical sample prevalence and characteristic data.**

***Response***

**You are right that this a limitation of our study (an inability to differentiate between those who self-identified as autistic from those who had a formal diagnosis) and we do not have a means to check survey data against patient's medical records. We have discussed this at greater length in the methodology and discussion sections of the manuscript.**

**Please note that comments relating to the self-reporting nature of the survey recur several times amongst the reviewers' comments, and in the interest of avoiding repetition we will direct back to the response to this first comment.**

***Location in Manuscript and Details of Changes***

**(Methods > Self-reported Autism Identification > 1st paragraph)**

**There were no means to identify which participants had a formal diagnosis of autism on their medical records (both among those self-reporting and not self-reporting autism); this issue is discussed in further detail in the Strengths and Weaknesses of the Study subsection of the Discussion.**

**(Discussion > Strengths and Weaknesses of the Study > 2nd paragraph)**

**Furthermore, the survey required adults to self-report long-term conditions, including autism, and we have no means to formally confirm their responses (such as an autism diagnosis) on their medical records. However, data on people self- or proxy-reporting autism via this approach has been previously described previously in the research literature,[25] and considering the high levels of autism underdiagnosis[13] and barriers to diagnostic assessment,[26] many adults self-reporting autism who lack a formal diagnosis may meet diagnostic criteria if they were to undergo diagnostic testing. Evidence of the similarities between clinically diagnosed and self-reporting autistic adults comes from Sturm et al,[27] using the RAADS-R[28] and RAADS-14[29] autism screening instruments, who reported 'few psychometric differences between diagnosed and self-identifyng (autistic) individuals.' Additionally. McDonald[30] found that in using the Autism Spectrum Identity Scale,[30] a measure of autism identity, both diagnosed and self-identifying autistic adults provided very similar results with respect to stigma, self-esteem, quality of life, and autism identity, Thus, whilst a level of bias may have been introduced through the self-reporting nature of autism in the context of the GPPS, research evidence suggests that those who self-identify as autistic without an accompanying diagnosis share important similarities to those with a formal**

**diagnosis. Considering the barriers to obtaining an autism diagnosis, high levels of underdiagnosis,[13] and the similarities that exist between these two groups, it is possible that a large sample of patients self-reporting as autistic provide a more accurate reflection of autistic adults in England compared to focussing solely on those with a formal diagnosis.**

**Comment #2**

***Comment***

**Abstract – Results: I assume that male and non-binary were discrete options, in which case I'd suggest rephrasing this to 'male or non-binary'.**

***Response***

**We have modified the abstract accordingly.**

***Location in Manuscript and Details of Changes***

**(Abstract > Results > 1st paragraph)**

**Adults self-reporting as autistic were more likely to be younger, male or non-binary, to identify as a gender different from their sex at birth, have a non-heterosexual sexual identity, be of White or mixed or multiple ethnic groups, non-religious, without caring responsibilities, unemployed, live in more deprived areas, and not smoke.**

**Comment #3**

***Comment***

**Introduction – I found the justification for this study a little hard to follow, as it feels like two different studies (characteristics/co-morbidities, and experiences of primary care) combined into one. The authors could make clearer the need for information about primary care experiences within this population.**

***Response***

**We have provided further detail to justify the need to better understand the primary care experiences of autistic people.**

***Location in Manuscript and Details of Changes***

**(Introduction > 1st paragraph)**

**Furthermore, diagnosed autistic people in the United Kingdom have significantly greater mortality rates compared to people without an autism diagnosis; 1.71 (95% CI 1.39-2.11) times the general population for people diagnosed with autism and not intellectual disability, and 2.83 (95% CI 2.33-3.43) times for people diagnosed with both autism and intellectual disability.[7] Relatedly, the life expectancy for people diagnosed with autism but not intellectual disability was found to be 6.14 years (95% CI 2.84-9.07) for men and 6.45 years (95% CI 1.37-11.57) for women.[7] All autistic people will access primary care during their lives, and for most health problems, the journey to diagnosis and treatment begins in this setting.[8] Thus, it is essential that primary care services are comprehensively meeting the needs of the autistic community.**

**Comment #4**

***Comment***

**Methods – it's not clear to me if there was any way to determine whether participants had a formal diagnosis of autism (whether or not they self-identify). To me, this is a limitation of the study as we don't know which participants had a formal diagnosis versus those who did not, which would be particularly novel and clinically relevant. As participant-level data was reported, could medical records be screened to identify those with a formal diagnosis? This seems particularly relevant as Table 1 implies that you might be able to distinguish between sub-types within each participant group.**

***Response***

***For detail related to how we have responded to the issue of self-reported autism, please see our response to Comment #1.***

***Location in Manuscript and Details of Changes***

***Please see our response to Comment #1.***

**Comment #5**

***Comment***

**I notice that one of the long-term health condition options was "another condition". Did the authors screen these responses to identify any relevant responses? I wonder if, in particular, self-identified autistic people might write something to the effect that they self-identify in this section, or might provide more context to their answer.**

***Response***

**Unfortunately, there is no further data available from the survey dataset recording what the respondent meant when they checked the 'another long-term condition or disability' item. We have now explained this in the relevant section of the manuscript.**

***Location in Manuscript and Details of Changes***

**(Methods > Demographic and Health Measures > 2nd paragraph)**

**Long-term health conditions listed as checklist options to the multiple-choice question included: Alzheimer's disease or other cause of dementia; Arthritis or ongoing problem with back or joints; Blindness or partial sight; A breathing condition such as asthma or chronic obstructive pulmonary disease (COPD); Cancer (diagnosis or treatment in the last 5 years); Deafness or hearing loss; Diabetes; A heart condition such as angina or atrial fibrillation; High blood pressure; Kidney or liver disease; A learning disability; A mental health condition; A neurological condition such as epilepsy; A stroke (which affects your day-to-day life); and Another long-term condition or disability (for this last option there was no accompanying free-text response space for the participant to specify the particular condition or disability that they were reporting).**

**Comment #6**

***Comment***

**Results**
**Page 10 – when reporting the demographic characteristics please clarify what the comparison group is; this is sometimes unclear (i.e. that people self-reporting as autistic were more likely to be male than those not reporting as autistic, rather than that this subgroup was more likely to be male than other gender identities?).**

*Response*

We have amended the text where we report the demographic characteristics so that we explicitly state the two groups which we are comparing.

*Location in Manuscript and Details of Changes*

(Results > Demographic Characteristics > 1st paragraph)

Compared to people not self-reporting as autistic, those who self-reported as autistic were more likely to describe their gender as male (ppd +17.4%) or non-binary (ppd +3.6%) and were also more likely to describe their gender as different from the sex registered at birth (ppd +5.9%) and to describe themselves as gay or lesbian (ppd +4.3%), bisexual (ppd +8.3%), or other (ppd +5.5%). Additionally, compared to people not self-reporting as autistic, those self-reporting as autistic were younger, with higher proportions in the 16–24-year (ppd +26.7%) and 25–34-year (ppd +14.9%) age groups. They were less likely to identify as being of Asian or Asian British (ppd -4.6%) or Black, Black British, Caribbean or African ethnicity (ppd -1.1%) and more likely to identify with no religion (ppd +15.7%). While adults self-reporting as autistic were less likely to report having parental responsibility for a child in their household (ppd -12.8%), they were slightly more likely to report having unpaid caring responsibilities for other persons and were more likely to report undertaking these roles for 50+ hours per week than those who did not self-report as autistic. In terms of employment status, people self-reporting as autistic were less likely to be in full-time work (ppd -21.7%), and more likely to be in full-time education (ppd +12.4%), unemployed (ppd +9.6%), or permanently sick or disabled (ppd +19.7%). They were also more likely to live in the most deprived neighbourhoods (ppd +8.7% for quintile 1, associated with the greatest level of deprivation).

<u>Comment #7</u>

*Comment*

Please add the ppd to the results reported for living in deprived neighbourhoods, for consistency (page 10, line 54).

*Response*

We have now added this information to the reported results.

*Location in Manuscript and Details of Changes*

(Results > Demographic Characteristics > 1st paragraph)

In terms of employment status, people self-reporting as autistic were less likely to be in full-time work (ppd -21.7%), and more likely to be in full-time education (ppd +12.4%), unemployed (ppd +9.6%), or permanently sick or disabled (ppd +19.7%). They were also more likely to live in the most deprived neighbourhoods (ppd +8.7% for quintile 1, associated with the greatest level of deprivation).

<u>Comment #8</u>

*Comment*

Page 11 – The sentence starting “Conditions where there are large differences…” (lines 23-30) is quite long and hard to follow. I’d suggest rephrasing and maybe breaking up into two sentences to give the examples and also make the point about there being greater differences between groups in younger people.

***Response***

**We agree this sentence is a bit long, so we have edited the text and also broken it up into two separate sentences.**

***Location in Manuscript and Details of Changes***

**(Results > Long-term Conditions > 2$^{nd}$ paragraph)**

**Conditions where there are large differences between participants with self-reported autism compared to those who do not report autism appear to be greater among younger age groups, showing some reduction in older age groups. Examples of such conditions include breathing conditions, learning disabilities, mental health conditions and neurological conditions.**

**Comment #9**

***Comment***

**The sensitivity analysis results reported in Table S3 (between different comparator groups) are interesting; I would like to see more discussion of these results although I appreciate the word count limits. In particular, these findings suggest some areas in which GP practices could make changes to improve the experiences of people with long-term conditions generally, which would also benefit autistic people, as well as areas in which autistic people may need specific adjustments or support.**

***Response***

**We agree that it is interesting that the sensitivity analysis indirectly shows that people with long-term conditions have poorer primary care experiences. However, this is not the principal focus of our manuscript and is a finding that has been described elsewhere before (e.g. Paddison, C. A., Saunders, C. L., Abel, G. A., Payne, R. A., Campbell, J. L., & Roland, M. (2015). Why do patients with multimorbidity in England report worse experiences in primary care? Evidence from the General Practice Patient Survey. *BMJ open*, *5*(3), e006172.)**

***Location in Manuscript and Details of Changes***

**(Results > Experiences of Primary Care > 5$^{th}$ paragraph)**

**Relatedly, Paddison and colleagues[19] have previously analysed the impact of long-term conditions more generally, including multimorbidity, on patient primary care experiences using GPPS data.**

**Comment #10**

***Comment***

**Discussion**
**I would like some more discussion about whether the characteristics findings are similar to those of other population-based studies in the UK which categorise participants based on clinical diagnosis. If the findings are broadly similar, it might suggest that using self-reported autism as an identifying category in research would allow greater inclusion of participants who might otherwise be excluded – the undiagnosed individuals identified in the study by O'Nions et al that the authors reference.**

***Response***

**We have now discussed our findings in comparison to the Adult Psychiatric Morbidity Survey (APMS), where autistic individuals are identified according to assessment by a clinically trained interviewer using the Autism Diagnostic Observation Schedule.**

***Location in Manuscript and Details of Changes***

**(Discussion > Strengths and Weaknesses in Relation to Other Studies > 1st paragraph)**

**The findings reported here should be compared with those from the Adult Psychiatric Morbidity Survey (APMS), a national survey of mental health and wellbeing among community-based adults in England.[20] Participants were identified with autism according to assessment with the Autism Diagnostic Observation Schedule (ADOS),21 undertaken by clinically trained interviewers, to ensure that the autism identification process was broadly similar to that used in clinical practice.[22] The estimated proportion of adults identified with autism from the combined 2007 and 2014 samples was around 0.8% (95% CI 0.5-1.3),[22] slightly lower than the 1.41% (95% CI 1.35 to 1.46) reported here. However, this estimate increased to 1.1% (95% CI 0.3-1.9) when accounting for adults with intellectual disability, who were not included in the APMS,[23] though are in the GPPS; thus, this latter estimate likely represents a more accurate figure when comparing with the GPPS respondent population. Furthermore, the sample identified with autism in the APMS did not demonstrate a clear pattern in age distribution, or employment status, in contrast to the GPPS findings reported here.**

**<u>Comment #11</u>**

***Comment***

**A key limitation that I think needs to be acknowledged is the lack of specificity in terms of participants being 'people who self-report as autistic' – i.e. not knowing whether these individuals are actually autistic (which I recognise is a subjective and contentious statement!). The authors note in Table 1 that people who self-report may not actually meet diagnostic criteria, and that there will be people who do not self-report who would meet diagnostic criteria, but I think there needs to be more consideration of how large these sub-groups could be. Overall I was left uncertain of whether or not the authors felt this sample represented a more accurate assessment of autistic people in the UK than a sample comprised of clinically diagnosed people. There could be some more discussion of why people choose to self-identify as autistic and whether they have similar characteristics to those who receive a formal diagnosis, perhaps in the introduction. I'm aware there is limited space, but this is a complicated issue and I think unfamiliar readers may need some more understanding of the context.**

***Response***

***For detail related to how we have responded to the issue of self-reported autism, please see our response to Comment #1.***

***Location in Manuscript and Details of Changes***

***Please see our response to Comment #1.***

**<u>REVIEWER TWO</u>**

**<u>Comment #12</u>**

***Comment***

**The authors utilise a very large sample drawn from a cross-sectional survey of adults registered with NHS GP surgeries in England, and demonstrate that there were some key differences in demographic features (e.g., higher rates of unemployment, higher rates of non-binary identity/differential gender identity to sex at birth) and health and social care experiences (e.g., higher rates of physical/mental health problems, reduced satisfaction in experiences interacting with services) between those who self-reported autism as a long-term health condition, as compared to those who did not in the survey. The overarching findings with regards to the experiences of autistic adults in England are important.**

***Response***

**Many thanks.**

***Location in Manuscript and Details of Changes***

**Not applicable – No changes required.**

**Comment #13**

***Comment***

**I had a couple of more minor specific points (please see below), however my main issue with the manuscript as it is currently framed is that the authors seem to place a big emphasis on the self reporting of autism here, when it is not clear that they were able to differentiate within this between those who self reported autism because they had received a formal diagnosis and those who self reported autism but had never received a formal diagnosis. This is noteworthy for two key reasons. Firstly, the authors imply in the summary box that what this study adds is evidence of experience of those self-reporting as autistic (beyond formal diagnosis), when it seems currently that the proportion of those within the 4,481 respondents who do not have a formal diagnosis is unknown. Therefore, the specific added value of this study needs to be reframed and/or clarified further (e.g., possibly this is more around the experiences of autistic adults, as compared to non autistic adults and those with other long term health conditions). Second, there is an increasing number of studies that do include self-identified autistic people without a formal diagnosis in research (for many of the reasons pointed out by the authors in their Introduction) and ongoing debates around whether or not this group are comparable to those who are formally diagnosed (e.g., there may be an over-representation of females in the self identified group, for instance) – the current framing of the paper feels a bit misleading, given this growth in research interest in self identification/diagnosis of autism.**

***Response***

***For detail related to how we have responded to the issue of self-reported autism, please see our response to Comment #1.***

**We have redrafted the 'What this study adds' section in response to the reviewers' comments.**

***Location in Manuscript and Details of Changes***

***Please see our response to Comment #1.***

**(Summary box > What this study adds)**

- **Compared to adults not self-reporting as autistic, adults self-reporting as autistic are at heightened risk of sensory, cognitive, neurological and mental health conditions.**

- **They experience difficulties accessing primary care services and are less likely to report their needs being met when they do. Practices need to improve service accessibility, particularly in terms of website design, ease of making appointments, and enabling patients to request a preferred GP.**

- **These findings can inform approaches to improving the primary care experiences of adults self-reporting as autistic.**

**<u>Comment #14</u>**

***Comment***

**Introduction**
**In the summary box, one of the bullet points is “Autism is underdiagnosed in adults.”, but I would feel a statement such as “Late diagnosis of autism (i.e., in adulthood) indicates that there is likely a notable group of adults who are undiagnosed.” [or the statement from the Introduction: “Adult general population surveys suggest that most adults with autism are not recognised (undiagnosed).”] Would be more accurate.**

***Response***

**We have removed the original bullet point, and replaced it with the second of the two options suggested by the reviewer.**

***Location in Manuscript and Details of Changes***

**(Summary box > What is already known on this topic)**

- **Adult general population surveys suggest that most adults with autism are not recognised (undiagnosed).**

**<u>Comment #15</u>**

***Comment***

**In the Introduction where the authors point out “In addition, many people may consider themselves to be autistic but who would not meet diagnostic thresholds if for autism assessed although they may have other significant, unidentified mental health conditions.” – it might be informative to add some detail around some of the underlying reasons for this (i.e., because those individuals are more likely to start off in mental health services, as is commonly reported in later diagnosed autistic females, for instance).**

***Response***

**We have provided some further detail in response to the possible reasons why some people may mistakenly consider themselves autistic.**

***Location in Manuscript and Details of Changes***

**(Introduction > 3$^{rd}$ paragraph)**

**In addition, many people may consider themselves to be autistic but who would not meet diagnostic thresholds if assessed for autism, although they may have other significant, unidentified mental health conditions. This may be in part due to autistic features overlapping with those of other neurodevelopmental conditions, such as learning disability and attention deficit hyperactivity disorder.[14]**

**<u>Comment #16</u>**

***Comment***

**Method**
**In the section 'self reported autism definition', it's not clear to me how the item option 'Autism or autism spectrum condition' to the multiple choice question 'Which, if any, of the following long-term conditions do you have?' could differentiate between adults with self-reported autism and adults who did not self report autism as per the total definition provided by the authors in Table 1. In other words, what was the specific question that ascertained self identification of autism (where there was no clinical diagnosis)?**

***Response***

***For detail related to how we have responded to the issue of self-reported autism, please see our response to Comment #1.***

***Location in Manuscript and Details of Changes***

***Please see our response to Comment #1.***

**<u>Comment #17</u>**

***Comment***

**In addition, were the authors able to also differentiate within the 'not self reporting as autistic' group between those who did have a clinical diagnosis, and those who did not (i.e., to compare those who self identify as autistic but do not have a clinical diagnosis of autism both to those who have a clinical diagnosis of autism, and to a general population sample of those who neither self identify as autistic nor have a clinical diagnosis of autism)?**

***Response***

***For detail related to how we have responded to the issue of self-reported autism, please see our response to Comment #1.***

***Location in Manuscript and Details of Changes***

***Please see our response to Comment #1.***

**<u>Comment #18</u>**

***Comment***

**Results/Discussion**
**The Results are interesting and stark, however the Discussion would benefit from greater emphasis on the interpretation and implications of the specific findings, as integrated more closely into the existing broader literature. There seemed to be a greater focus here on strengths/limitations of the work (though this did not touch upon the issue outlined above regarding the lack of clarity over who is included in the self reporting as autistic group'). Particularly, expansion on clinical/policy implications to address the health and social care experiences reported by autistic adults would be critical.**

***Response***

**We have brought in further reference to the published literature into our discussion section, as well as provided greater emphasis on the policy implications of the analysis.**

*Location in Manuscript and Details of Changes*

The data reported here show that people self-reporting as autistic were more likely to report taking a variety of different actions prior to attempting to make a general practice appointment (Table 4). This is consistent with findings from autistic focus groups, who describe feeling 'reluctant to seek help' and 'that they only access primary care as a last resort.[8]' A survey of autistic adults has previously idntified barriers to visiting their GP, including being unsure if their symptoms warrant a visit, difficulty making appointments via telephone, not feeling understood, difficulty communicating with their doctor, and the waiting room environment.[36] These findings are valuable in informing public health interventions targeting this group; knowing that people self-reporting as autistic are more likely to speak to a pharmacist or seek advice from a friend or family member underlines a need to public health interventions are informed by such frequently used pathways. Furthermore, there is a need to reflect as to why adults self-reporting as autistic are more likely to take these other actions prior to making a general practice appointment – for instance, is this in part a reflection of their difficulties in navigating their GP practice's website? One goal of the NHS Long Term Plan[9] is to improve staff understanding of the needs of autistic people, and the survey analysis reported here can help inform policy initiatives that are sensitive and responsive to the needs of the autistic community. One such approach could be specialized clinics for autistic people; evidence from the United States supports such approaches, where clinician continuity is prioritised, as well as patient's sensory needs.[37]

<u>REVIEWER THREE</u>

<u>Comment #19</u>

*Comment*

Thank you for the opportunity to review and comment on the report of this interesting study examining the characteristics and primary care experiences of people who self-report at autistic. The work is of relevance since it reports research on an underserved UK population. The research itself is based on GP Patient Survey data – this being a large routine survey undertaken and overseen by the NHS. In general, I found the work to be excellent – highly relevant and potentially important identifying gaps in the primary care system – particularly in respect of this vulnerable population.

*Response*

Many thanks.

*Location in Manuscript and Details of Changes*

Not applicable – No changes required.

<u>Comment #20</u>

*Comment*

The authors draw on GPPS data to identify the characteristics of individuals self-reporting as autistic, comparing this with other survey respondents. Several distinctive characteristics are identified.

*Response*

Many thanks.

***Location in Manuscript and Details of Changes***

**Not applicable – No changes required.**

**<u>Comment #21</u>**

***Comment***

**My main concern with the study relates to table 3 which I find confusing. The table presents a number of comparisons for individuals who self-reported as autistic as compared to those who did not, presenting findings as adjusted odds ratios. However, those reporting as autistic sometimes had increased or decreased prevalence of long-term health conditions but the adjusted odds ratios are always positive and >1 despite the reduced prevalence - for example in respect of diabetes or heart conditions. I presume this has to do with the “adjustments” being made in the analysis but in the absence of univariate associations it is difficult to understand this somewhat surprising finding. This may be my lack of understanding and statistical expertise, but surely one would expect the adjusted odds ratio to be less <1 where the prevalence was reduced against the comparator group.**

***Response***

**The reviewer is correct that the positive adjusted odds ratios (even when the unadjusted prevalence is lower), are a consequence of adjusting for age, gender, deprivation, and ethnicity, which we have now explained further in the manuscript text.**

***Location in Manuscript and Details of Changes***

**(Results > Long-term Conditions > 1st paragraph)**

**It should be noted that the participants self-reporting as autistic were more likely to be younger, male or non-binary, have a greater level of neighbourhood deprivation, and be of White ethnicity, underlining the importance for adjusted analyses. After adjustment, all 16 conditions had higher odds of occurring among people self-reporting as autistic than those who did not.**

**<u>Comment #22</u>**

***Comment***

**The authors do consider the issue of self-reporting against confirmed diagnosis (in 7/47 etc.) but I would like to see some more detail on the implications of self-reporting and this reporting bias in respect of the results presented.**

***Response***

***For detail related to how we have responded to the issue of self-reported autism, please see our response to Comment #1.***

***Location in Manuscript and Details of Changes***

***Please see our response to Comment #1.***

**<u>Comment #23</u>**

***Comment***

**Data for GPPS 2002 was used to undertake this study – under what circumstances and arrangements was this data made available to the study team?**

***Response***

**We have added further detail in the acknowledgements section explaining how the data was made available to the study team.**

***Location in Manuscript and Details of Changes***

**(Acknowledgements)**

**We would like to thank Geraldine Egboche and Vicki Bolton at Ipsos MORI for their kind support with this work, with respect to facilitating our application for the survey data required to conduct the analysis reported in this manuscript. Please note that the Ipsos MORI team had no involvement in the analysis, interpretation, or writing of this manuscript.**

**Comment #24**

***Comment***

**Is 9/48 correct in terms of grammar/presentation**

***Response***

**We have amended this sentence to make it clearer to the reader.**

***Location in Manuscript and Details of Changes***

**(Methods > Patient Experience Measures > 1st paragraph)**

**The survey also included a series of questions relating to participant's experiences of primary care services, with corresponding Likert scale question response options.**

**Comment #25**

***Comment***

**10/40 is this about the marginal probability of reporting of each long-term condition – is something missing in this sentience?**

***Response***

**We have amended this sentence to make it clearer.**

***Location in Manuscript and Details of Changes***

**The marginal probability of each long-term condition according to autism status and age group was calculated and are presented graphically.**

**Comment #26**

***Comment***

**10/58 What is the potential for completion of the questionnaire by carers on behalf of autistic respondents?**

***Response***

**We agree that this is possible, though data on such proxy completion was not collected. We nevertheless cover this issue now in the Methods and Discussion sections of the manuscript.**

***Location in Manuscript and Details of Changes***

**(Methods > Self-reported Autism > 2nd paragraph)**

**Considering this, it is possible that some surveys, particularly from those with a learning disability, may have been completed with support from a carer; however, no data was collected with respect to whether any surveys were completed by either assistance from a proxy, or the proxy completing the survey on behalf of the patient.**

**(Discussion > Strengths and Weaknesses of the Study > 3rd paragraph)**

**It is also possible that some adults self-reporting as autistic may have received support from a carer in undertaking the survey, particularly those with co-occurring learning disability, which may have impacted on the responses provided. However, no data was collected with respect to whether any surveys were completed by a proxy.**

<u>**Comment #27**</u>

***Comment***

**11/21 – typo “conditions”**

***Response***

**Typo addressed.**

***Location in Manuscript and Details of Changes***

**(Results > Long-term Conditions > 2nd paragraph)**

**Figure 2 depicts differences in the marginal probability of each long-term condition between those who self-reported as autistic and those who did not by age.**

<u>**Comment #28**</u>

***Comment***

**12/34-38 notes that, for several questions, no significant differences were found between items. The authors then go on to focus on areas where substantial differences were observed. However, overall experience of GP care is a key national metric which I think should be specifically noted – there were no difference between autistic and non-autistic participants as per table 4.**

***Response***

**We agree that this is an important and relevant finding, and have now included it within the manuscript text in addition to Table 4.**

***Location in Manuscript and Details of Changes***

**(Results > Experiences of Primary Care > 2nd paragraph)**

**However, no significant difference was reported between participant’s self-reporting as autistic compared to those not self-reporting as autistic with respect to their overall experience of their GP practice.**

**(Discussion > Principal Findings > 1st paragraph)**

**Whilst no significant difference was observed with respect to their overall experience of their GP practice, adults self-reporting as autistic reported lower confidence in healthcare professionals, and were less likely to report their needs being met.**

**Comment #29**

***Comment***

**13/3 – typo**

***Response***

**We have addressed this typo.**

***Location in Manuscript and Details of Changes***

**(Results > Experiences of Primary Care > 3rd paragraph)**

**No significant differences were identified with respect to being able to see or speak to their preferred GP (compared to those who did not self-report as autistic).**

**Comment #30**

***Comment***

**15/8 – typo**

***Response***

**Typo addressed.**

***Location in Manuscript and Details of Changes***

**(Discussion > Implications for Research, Clinical Practice, and Policy > 1st paragraph)**

**Furthermore, there is a need to reflect as to why adults self-reporting as autistic are more likely to take these other actions prior to making a general practice appointment – for instance, is this in part a reflection of their difficulties in navigating their GP practice's website?**

**Comment #31**

***Comment***

**Overall, I thought this was an excellent and well-presented piece of work but with some important issues, particularly in respect of table 3.**

***Response***

**Many thanks – We have endeavoured to respond to your requested amendments, including relating to Table 3 (See response to Comment #21).**

***Location in Manuscript and Details of Changes***

**Not applicable – No changes required.**

**REVIEWER FOUR**

**Comment #32**

***Comment***

**In this population-based, cross-sectional study, Tromans and colleagues aimed to identify the characteristics and primary care experiences of people who self-report as autistic. Of 623,157 responders to the survey, 4,481 self-reported as autistic. Those self-reporting as autistic were more likely to be younger, male, non-binary, to identify as a gender different from their sex at birth, have a non-heterosexual sexual identity, be of White or mixed or multiple ethnic groups, non-religious, without caring responsibilities, unemployed, live in more deprived areas, and not smoke. In an adjusted multivariable logistic regression model, the authors further investigated chronic conditions that were more prevalent among adults self-reporting as autistic.**
**This is an important study covering an overlooked population. Part of the results of the study will be useful to better understand the primary care needs and experiences of people self-reporting as autistic. However, other aspects of the study are not interpretable. The authors may want to consider the following comments.**

***Response***

**Many thanks – We have endeavoured to address all of your comments.**

***Location in Manuscript and Details of Changes***

**Not applicable – No changes required.**

**Comment #33**

***Comment***

**1. The aim of the study is clearly descriptive, and the first part of the study (i.e., Table 2) uses methods to describe the characteristic patterns of people self-reporting as autistic. However, the rationale of the adjusted logistic regression model is not clear. An adjusted model aims to address potential confounding in the causal research domain, which, however, was not the aim of the study. How can the adjusted odds ratios be interpreted? What does it mean to show effect estimates in a hypothetical world where participants have the same age, gender, deprivation, and ethnicity? Why adjust for these factors? Further, adjusting for some of these factors, particularly ethnicity, may result in structural biases (http://dx.doi.org/10.1093/epirev/mxad010). The authors should be clear about the research question that is associated with this part of the study. I recommend staying in the descriptive domain and use, for example, pattern recognition or bootstrap techniques to describe the ranking or importance of certain variables. Alternatively, univariate odds ratios can be shown.**

***Response***

**We understand that this is about describing the profile of a population; this is why we present a lot of descriptive profiling. However, one of our research questions is to understand whether people who self-report as autistic have particular primary care needs or experiences, and in order to focus in on that we also present adjusted analyses to address the research question as to whether their experiences are associated with their autistic status rather than other demographic characteristics. We have now amended the manuscript to clarify how the adjusted analyses relate to a specific research question.**

***Location in Manuscript and Details of Changes***

**(Methods > Statistical Analysis > 2$^{nd}$ and 3$^{rd}$ paragraphs)**

**. Adjusted analyses were carried out to examine the associations between other long-term conditions and self-reporting as autistic, involving logistic regression models incorporating probability weights and adjusting for age, ethnicity, gender and area-level deprivation (IMD quintile) were fitted to return adjusted odds ratios (aOR) with 95% CIs and p-values.**

**Adjusted analyses (as described above) were also undertaken to compare the primary care experiences of adults who self-report as autistic with those who do not, producing weighted percentages and aORs using logistic regression.**

**Comment #34**

***Comment***

**2. Part of the conclusions of the authors, in particular when calling for public health interventions, require a proper causal framework. Such conclusions are, therefore, not supported by the provided data.**

***Response***

**We have adjusted our language in the discussion to clarify that this is a potential policy implication which warrants further longitudinal data in order to establish causality.**

***Location in Manuscript and Details of Changes***

**(Discussion > Strengths and Weaknesses of the Study > 2nd paragraph)**

**Whilst reporting association between self-reporting as autistic with long-term conditions and primary care experiences is informative, data from the 2022 GPPS is cross-sectional in nature, and longitudinal data is required to establish causality**

**Comment #35**

***Comment***

**3. The authors should be more specific on how age, ethnicity, gender, and deprivation score were used in the logistic regression model.**

***Response***

**Thank you, we have now described in the manuscript how the adjustment variables were coded and that they were entered into the regression model as categorical variables.**

***Location in Manuscript and Details of Changes***

**(Methods > Statistical Analysis > 2nd paragraph)**

**Age (16-24 years; 25-34; 35-44; 45-54; 55-64; 65-74; 75 or over), ethnicity (White; Mixed or Multiple ethnic groups; Asian or Asian British; Black, Black British, Caribbean, or African; Other ethnic group), gender (Female; Male; Non-binary; Prefer to self-describe), and area-level deprivation/ IMD quintile (1 [most deprived]; 2; 3; 4; 5 [least deprived]) were all entered into the regression model as categorical variables.**

**Comment #36**

***Comment***

**4. I may have missed it, but did the study have ethical approval?**

***Response***

**We have now provided an ethics statement within the Methods section of the manuscript.**

***Location in Manuscript and Details of Changes***

**(Methods > Ethical Approval > 1st paragraph)**

**Ipsos MORI were advised by the Central Office for Research Ethics Committee that the GPPS is a service evaluation rather than pure research, and for this reason the survey does not require formal research ethics approval.[18]**

**Comment #37**

***Comment***

**5. The authors mention two people from Ipsos for their support. What was their support, and how was Ipsos involved in the study?**

***Response***

**We have provided further details in the Introduction and Acknowledgements sections as to the role of Ipsos MORI.**

***Location in Manuscript and Details of Changes***

**(Introduction > 4th paragraph)**

**The General Practice Patient Survey (GPPS), conducted by Ipsos MORI, is an NHS England-funded annual national cross-sectional survey of adults aged 16 and over who have been registered with a GP practice in England for at least 6 months, to provide evidence to support healthcare improvement for process measures of care quality.**

**(Acknowledgements)**

**We would like to thank Geraldine Egboche and Vicki Bolton at Ipsos MORI for their kind support with this work, with respect to facilitating our application for the survey data required to conduct the analysis reported in this manuscript. Please note that the Ipsos MORI team had no involvement in the analysis, interpretation, or writing of this manuscript.**

**Comment #38**

***Comment***

**6. I may have missed it, but what statistical software was used to analyze the data?**

***Response***

**We have added a statement in the Statistical Analysis section of the Methods section relating to the statistical software used.**

***Location in Manuscript and Details of Changes***

**(Methods > Statistical Analysis > 1st paragraph)**

**All analyses were performed using Stata version 17.**

**Comment #39**

***Comment***

**7. As the authors report P values (which is unnecessary), they should specify the alpha level and whether the reported P values are one or two-tailed.**

***Response***

**Thank you for this comment – considering that confidence intervals provide a much more nuanced insight into precision and differences between groups. As a result, we have removed P-values from the corresponding Tables in order to simplify the presentation of the results.**

***Location in Manuscript and Details of Changes***

**(P-values have been removed from tables in both the main manuscript and supplementary material).**

**Comment #40**

***Comment***

**8. I suggest removing the number of people who self-reported as autistic from the study's title.**

***Response***

**We have changed the title as requested.**

***Location in Manuscript and Details of Changes***

**(Title)**

**The characteristics and primary care experiences of people who self-report as autistic: a probability sample survey of adults registered with primary care services in England**

**Comment #41**

***Comment***

**9. I suggest removing the statement that this is the largest study on this topic from the What This Study Adds section**

***Response***

**We have removed this statement.**

***Location in Manuscript and Details of Changes***

**(Summary Box > What This Study Adds)**

***Related Sentence Removed from Manuscript***

**Comment #42**

***Comment***

**10. A flow chart will help readers to understand the final study sample.**

***Response***

**We have included a flow chart summarising how the final study sample was arrived at.**

***Location in Manuscript and Details of Changes***

**(Results > Frequency of Self-reported Autism > 1$^{st}$ paragraph)**

**Figure 1 shows a flow chart illustrating how the final analysis study population was attained.**

***New Figure added to manuscript***

**VERSION 2 – REVIEW**

| | |
|---|---|
| **REVIEWER NAME** | ***Hull, Laura*** |
| **REVIEWER AFFILIATION** | University College London |
| **REVIEWER CONFLICT OF INTEREST** | Na |
| **DATE REVIEW RETURNED** | 06-Aug-2024 |

| | |
|---|---|
| **GENERAL COMMENTS** | My thanks to the authors for addressing my comments; I have one remaining suggestion which I believe will improve the interpretation of their conclusions, as well as identifying a minor typo.<br><br>I appreciate the authors' addition of more information regarding the status of formally diagnosed or undiagnosed autism within their sample, which helps to contextualise their work. However I felt the authors could include a brief additional discussion of the implications of not distinguishing between diagnosed and non-diagnosed autistic people in this sample. For instance, what are the consequences if the rates of autism are under or over estimated in this study?<br><br>There is a potential typo in Page 7, end of Paragraph 1 ("the life expectancy…was found to be 6.14 years" – presumably this is 6.14 years shorter?). |

| | |
|---|---|
| **REVIEWER NAME** | ***Oakley, Bethany*** |
| **REVIEWER AFFILIATION** | King's College London |
| **REVIEWER CONFLICT OF INTEREST** | Na |
| **DATE REVIEW RETURNED** | 16-Aug-2024 |

| | |
|---|---|
| **GENERAL COMMENTS** | I thank the authors for the revisions made in response to reviewer comments, which now make the approaches of the study more transparent, and also better emphasise the importance of these approaches.<br>I have a couple of minor points for a final version of this manuscript.<br><br>1. In the abstract, the opening line ("Little is known about adults who self-report as autistic") still provides the immediate impression that this is more focused on self-identified (rather than diagnosed) autistic people. While this is clarified well now in the later sections of the paper, this opening line still does feel a little misleading. Perhaps |

| | |
|---|---|
| | a small edit (e.g., "Little is known about adults who self-report as autistic (including those with and without a formal diagnosis)") would address this right up front. It may also be helpful to briefly comment on the areas in which little is known to provide the context for this paper. Autism research progress (while still having a long way to go) has increased substantially in the past decade - so, is the key thing here that little is known about the adult outcomes and healthcare experiences of autistic adults?<br><br>2. I thank the authors for addressing my #Comment 15, though I apologise that my original phrasing of this comment has led to a slight misinterpretation. What I had hoped to convey here was that it would be interesting for the authors to briefly comment on some of the potential limits/limitations of existing diagnostic instruments and pathways that mean that some people who consider themselves autistic would not meet diagnostic thresholds. While it's true that some features may be better attributed in the end to other conditions with overlapping features (as the authors highlight now), we also know that for some autism presentations, our existing tools are not always accurate/optimal (e.g., in autistic women, and in reflecting developmental changes in autism features when diagnosis is made during adulthood). In other words, and hopefully more supportive of the authors' original argument, it's not only about people mistakenly considering themselves autistic, but actually being autistic but just not always meeting thresholds on our existing tools. |

| | |
|---|---|
| **REVIEWER NAME** | ***Campbell, John*** |
| **REVIEWER AFFILIATION** | University of Exeter, Primary Care |
| **REVIEWER CONFLICT OF INTEREST** | Previously published (many years ago) research with Saunders |
| **DATE REVIEW RETURNED** | 25-Jul-2024 |

| | |
|---|---|
| **GENERAL COMMENTS** | Thank you for opportunity to review the revised material. A major revision has been undertaken. The 4 sets of reviewer comments have been comprehesively responded-to as far as I can see. Certainly, my own comments have been addressed in full and I would recommend publication of the material as a useful and interesting addition to teh relevant reserach literature, providing new insights on a particularly vulnerable group of individuals and their experience of primary healthcare provision. |

**VERSION 2 – AUTHOR RESPONSE**

**REVIEWER ONE**

**Comment #4**

***Comment***

**My thanks to the authors for addressing my comments; I have one remaining suggestion which I believe will improve the interpretation of their conclusions, as well as identifying a minor typo.**

***Response***

**Many thanks for kindly reviewing our manuscript twice and for your helpful comments.**

***Location in Manuscript and Details of Changes***

**N/A – No changes required.**

**Comment #5**

***Comment***

**I appreciate the authors' addition of more information regarding the status of formally diagnosed or undiagnosed autism within their sample, which helps to contextualise their work. However, I felt the authors could include a brief additional discussion of the implications of not distinguishing between diagnosed and non-diagnosed autistic people in this sample. For instance, what are the consequences if the rates of autism are under or overestimated in this study?**

***Response***

**Thank you for identifying this potential issue, though it is important to acknowledge that the GPPS is not a prevalence survey, but rather intended to report on how patients feel about their GP practice. We have discussed the potential adverse implications if it were interpreted in this way, and linked this to our previous comments about asking respondents to either confirm if they have a clinical diagnosis for the long-term conditions they report having, or cross-checking patient GPPS responses against their electronic healthcare records.**

***Location in Manuscript and Details of Changes***

***(Discussion > Principal findings > 3$^{rd}$ paragraph)***

**The GPPS is not intended as a prevalence survey, but rather aims to report on how patients feel about their GP practice, with a view to improving their healthcare. Thus, the estimated proportion of individuals self-reporting as autistic in our analysis should not be interpreted as a definitive prevalence estimate, as doing so could lead to misallocation of healthcare resources.**

**Comment #6**

***Comment***

**There is a potential typo in Page 7, end of Paragraph 1 ("the life expectancy…was found to be 6.14 years" – presumably this is 6.14 years shorter?).**

***Response***

**Thank you for identifying this typo – we have amended the sentence accordingly.**

***Location in Manuscript and Details of Changes***

***(Introduction > 1$^{st}$ paragraph)***

**Relatedly, the life expectancy for people diagnosed with autism but not intellectual disability was found to be shorter by 6.14 years (95% CI 2.84-9.07) for men and 6.45 years (95% CI 1.37-11.57) for women.[7]**

**REVIEWER TWO**

**<u>Comment #7</u>**

***Comment***

**I thank the authors for the revisions made in response to reviewer comments, which now make the approaches of the study more transparent, and also better emphasise the importance of these approaches.**
**I have a couple of minor points for a final version of this manuscript.**

***Response***

**Many thanks for kindly reviewing our manuscript twice and for your helpful comments.**

***Location in Manuscript and Details of Changes***

**N/A – No changes required.**

**<u>Comment #8</u>**

***Comment***

**In the abstract, the opening line ("Little is known about adults who self-report as autistic") still provides the immediate impression that this is more focused on self-identified (rather than diagnosed) autistic people. While this is clarified well now in the later sections of the paper, this opening line still does feel a little misleading. Perhaps a small edit (e.g., "Little is known about adults who self-report as autistic (including those with and without a formal diagnosis)") would address this right up front. It may also be helpful to briefly comment on the areas in which little is known to provide the context for this paper. Autism research progress (while still having a long way to go) has increased substantially in the past decade - so, is the key thing here that little is known about the adult outcomes and healthcare experiences of autistic adults?**

***Response***

**We have amended the abstract as requested, as well as making reference in the introduction to the comparative lack of focus on autistic adults.**

***Location in Manuscript and Details of Changes***

***(Abstract > Objectives)***

**Little is known about adults who self-report as autistic (including those with and without a formal diagnosis).**

***(Introduction > 2$^{nd}$ paragraph)***

**However, there is a lack of autism research focused on adults, with a Web of Science topic search demonstrating that in 2020, only around 21% of autism publications contained that ‘adult*’ search term.[11]**

**<u>Comment #9</u>**

***Comment***

**I thank the authors for addressing my #Comment 15, though I apologise that my original phrasing of this comment has led to a slight misinterpretation. What I had hoped to convey here was that it would be interesting for the authors to briefly comment on some of the**

**potential limits/limitations of existing diagnostic instruments and pathways that mean that some people who consider themselves autistic would not meet diagnostic thresholds. While it's true that some features may be better attributed in the end to other conditions with overlapping features (as the authors highlight now), we also know that for some autism presentations, our existing tools are not always accurate/optimal (e.g., in autistic women, and in reflecting developmental changes in autism features when diagnosis is made during adulthood). In other words, and hopefully more supportive of the authors' original argument, it's not only about people mistakenly considering themselves autistic, but actually being autistic but just not always meeting thresholds on our existing tools.**

***Response***

**We agree that as well as overlapping features, the limitations of autism diagnostic tools should also be mentioned, and have now added further information to the introductory section with this in mind.**

***Location in Manuscript and Details of Changes***

***(Introduction > 3rd paragraph)***

**Additionally, it is important to recognise the limitations of current autism diagnostic tools, particularly in certain groups, such as women and girls.[16]**

**REVIEWER THREE**

**Comment #10**

***Comment***
**Thank you for opportunity to review the revised material. A major revision has been undertaken. The 4 sets of reviewer comments have been comprehensively responded-to as far as I can see. Certainly, my own comments have been addressed in full and I would recommend publication of the material as a useful and interesting addition to the relevant research literature, providing new insights on a particularly vulnerable group of individuals and their experience of primary healthcare provision.**

***Response***

**Many thanks for kindly reviewing our manuscript twice and for your helpful comments.**

***Location in Manuscript and Details of Changes***

**N/A – No changes required.**

## VERSION 3 – REVIEW

| | |
|---|---|
| **REVIEWER NAME** | ***Hull, Laura*** |
| **REVIEWER AFFILIATION** | University College London |
| **REVIEWER CONFLICT OF INTEREST** | Na |
| **DATE REVIEW RETURNED** | 28-Aug-2024 |

| | |
|---|---|
| **GENERAL COMMENTS** | My thanks to the authors for this further round of changes; I am now satisfied with the manuscript. |

| | |
|---|---|
| **REVIEWER NAME** | ***Oakley, Bethany*** |
| **REVIEWER AFFILIATION** | King's College London |
| **REVIEWER CONFLICT OF INTEREST** | Na |
| **DATE REVIEW RETURNED** | 27-Aug-2024 |

| | |
|---|---|
| **GENERAL COMMENTS** | I thank the author for addressing my comments, and wish them all the best with the manuscript. |
